# A Spatiotemporally Controlled Gene‐Regulation Strategy for Combined Tumor Therapy Based on Upconversion Hybrid Nanosystem

**DOI:** 10.1002/advs.202405640

**Published:** 2024-08-29

**Authors:** Fang Wang, Zechao Liu, Yuechen Liu, Jiayi Zhang, Weizhe Xu, Bei Liu, Zhaogang Sun, Hongqian Chu

**Affiliations:** ^1^ Translational Medicine Center Beijing Chest Hospital Capital Medical University 9 Beiguan Street Beijing 101149 China; ^2^ College of Science Minzu University of China 27 Zhongguancun South Avenue Beijing 100081 China

**Keywords:** enzyme‐activated, gene therapy, light‐controlled, photodynamic therapy, upconversion nanoparticles

## Abstract

The lack of precise spatiotemporal gene modulation and therapy impedes progress in medical applications. Herein, a 980 nm near‐infrared (NIR) light‐controlled nanoplatform, namely URMT, is developed, which can allow spatiotemporally controlled photodynamic therapy and trigger the enzyme‐activated gene expression regulation in tumors. URMT is constructed by engineering an enzyme‐activatable antisense oligonucleotide, which combined with an upconversion nanoparticle (UCNP)‐based photodynamic nanosystem, followed by the surface functionalization of triphenylphosphine (TPP), a mitochondria‐targeting ligand. URMT allows for the 980 nm NIR light‐activated generation of reactive oxygen species, which can induce the translocation of a DNA repair enzyme (namely apurinic/apyrimidinic endonuclease 1, APE1) from the nucleus to mitochondria. APE1 can recognize the basic apurinic/apyrimidinic (AP) sites in DNA double‐strands and perform cleavage, thereby releasing the functional single‐strands for gene regulation. Overall, an augmented antitumor effect is observed due to NIR light‐controlled mitochondrial damage and enzyme‐activated gene regulation. Altogether, the approach reported in this study offers high spatiotemporal precision and shows the potential to achieve precise and specific gene regulation for targeted tumor treatment.

## Graphical abstract

A near‐infrared (NIR) light‐controlled nanosystem is developed that allows spatiotemporally controlled photodynamic therapy and further triggers the enzyme‐activated gene expression regulation in tumors. The nanosystem is constructed by upconversion nanoparticles, photosensitizer, enzyme‐activatable antisense oligonucleotide, mitochondria targeting molecules, which provide an augmented antitumor effect because of NIR light‐induced mitochondrial photodamage and enzyme‐activated gene regulation.

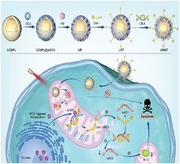

## 1 Introduction

Photodynamic therapy (PDT) is an attractive tumor treatment modality approved, which combines light, oxygen, and photosensitizers (PSs) to generate cytotoxic reactive oxygen species (ROS).^[^ ^1^, ^2^, ^3^ ^]^ PDT presents inherent advantages such as high selectivity and noninvasiveness;^[^ ^4^, ^5^, ^6^ ^]^ however, its clinical application remains a challenge. For example, PDT exhibits limited effectiveness in treating tumors developed in the depth of the body because of the low penetration depth of the excitation light for PSs.^[^ ^7^, ^8^ ^]^ Additionally, the hypoxic nature of various solid tumors limits the therapeutic effect of PDT.^[^ ^9^, ^10^, ^11^ ^]^ Mitochondria, the energy factories of cells, are considered crucial subcellular target for many PSs employed in PDT.^[^ ^12^, ^13^, ^14^ ^]^ However, despite notable progress, the effect of PDT on mitochondria alone has been insufficient, necessitating the exploration of combinations of PDT with other therapeutic approaches.

Gene therapy possesses tremendous potential for tumor treatment. MicroRNAs (miRNAs) are small, single‐stranded, and evolutionarily conserved RNA molecules, which play an important role in preventing the translation of specific messenger RNA (mRNA).^[^ ^15^ ^]^ The dysregulated of miRNA expression is associated with various diseases.^[^ ^16^, ^17^ ^]^ The molecular functions of individual miRNAs can be determined by inhibiting their activity and identifying subsequent changes in corresponding mRNA or protein levels, along with other phenotypic changes.^[^ ^18^ ^]^ Therefore, miRNAs are considered promising therapeutic targets in tumor treatment.^[^ ^19^, ^20^ ^]^ miR‐21 is a kind of microRNA frequently over‐expressed in a wide range of human cancers.^[^ ^21^, ^22^ ^]^ Various studies showed that miR‐21 plays a crucial role in promoting tumorigenesis, characterized by increased proliferation, decreased apoptosis, enhanced invasion and increased metastatic potential.^[^ ^23^, ^24^, ^25^, ^26^ ^]^ The reduction or silencing of miR21 can reduce the level of Bcl‐2 protein, activate the mitochondrial apoptosis pathways, and promote the apoptosis of tumor cells.^[^ ^27^, ^28^ ^]^ Thus, miR‐21 is considered as an important target for cancer therapy. However, owing to the leakiness of even tissue‐specific promoters, ensuring target tissue‐specific expression of the therapeutic transgene while minimizing off‐target effects remains a challenge.^[^ ^18^ ^]^ Additionally, traditional gene delivery systems lack spatiotemporal specificity and cannot activate genes at specific lesion sites, severely hindering the efficacy of gene therapy.^[^ ^29^, ^30^, ^31^ ^]^

Recently, light‐controlled techniques have gained a lot of attention due to their exceptional spatiotemporal accuracy.^[^ ^32^, ^33^, ^34^ ^]^ Particularly in the neuroscience and other life sciences fields, the development of light‐controlled chemical reactions or drug release systems has brought about revolutionary advancements.^[^ ^35^, ^36^, ^37^ ^]^ In recent years, owing to its remarkable programmability and excellent biocompatibility, DNA has emerged as a versatile tool for engineering intelligent nanosystems.^[^ ^38^, ^39^, ^40^, ^41^, ^42^ ^]^ Various well‐defined, light‐controlled DNA nanodevices have been constructed to achieve comprehensive effects in tumor therapy.^[^ ^43^, ^44^, ^45^ ^]^ However, the reliance on ultraviolet (UV) or blue light in these systems considerably hinders their application because of limitations such as poor tissue penetration and phototoxicity.^[^ ^46^, ^47^, ^48^ ^]^ Additionally, although near‐infrared (NIR) light‐activated gene therapy demonstrates deep penetration capabilities,^[^ ^49^, ^50^, ^51^, ^52^, ^53^, ^54^ ^]^ the dependence on exogenous activation hinders its clinical implementation.

**Figure 1:**
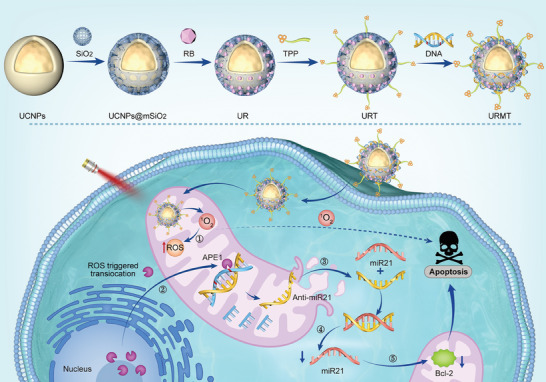
Schematic showing the working principle of the nanosystem for mitochondrial targeted tumor gene therapy based on photodynamic activated APE1 translocation. Apurinic/apyrimidinic endonuclease 1: APE1; UCNPs: upconversion nanoparticles; UR: UCNPs@mSiO_2_‐RB; URT: UCNPs@mSiO_2_‐RB‐TPP; URMT: URT packed with dsDNA containing basic AP sites.

Herein, we constructed a novel upconversion nanoparticle (UCNP)‐based hybrid nanosystem for light‐activated and enzyme‐triggered PDT/gene combinational tumor therapy. The nanosystem, termed URMT, is assembled by integration of PSs with a rationally designed oligonucleotide (hereby referred to as AM), which can be activated by apurinic/apyrimidinic endonuclease 1 (APE1) (**Figure** **1**). These components were assembled on the surface of the UCNPs, following which a mitochondrial targeting ligand, namely triphenylphosphine (TPP), was introduced for precise targeting.^[^ ^55^ ^]^ AM was synthesized by hybridizing the antisense oligonucleotide of anti‐miR21 with the complementary DNA stand of miR21 containing two AP sites (Figure S1, Supporting Information). This methodology employs photon upconversion nanotechnology to achieve PDT, promoting subcellular translocation of APE1.^[^ ^56^, ^57^, ^58^ ^]^ Additionally, APE1 translocation from the nucleus to mitochondria facilitated enhanced enzymatic cleavage of the AP sites in the miR21 double strand, subsequently releasing anti‐miR21 into the cytoplasm, which led to the downregulation of miR21 expression. The downregulation of miR21 further downregulated Bcl‐2 to initiate cell apoptosis.^[^ ^59^, ^60^ ^]^ This approach combines 980 nm light‐triggered mitochondrial damage with photo‐activatable to achieve a combinational tumor treatment strategy. Notably, URMT, activated by NIR light and endogenous enzymes, provides highly specific and precise spatiotemporal control for PDT and gene combination therapy, while ensuring a high level of safety.

## 2 Results and Discussion

### 2.1 Synthesis and Characterization of URMT

**Figure 2:**
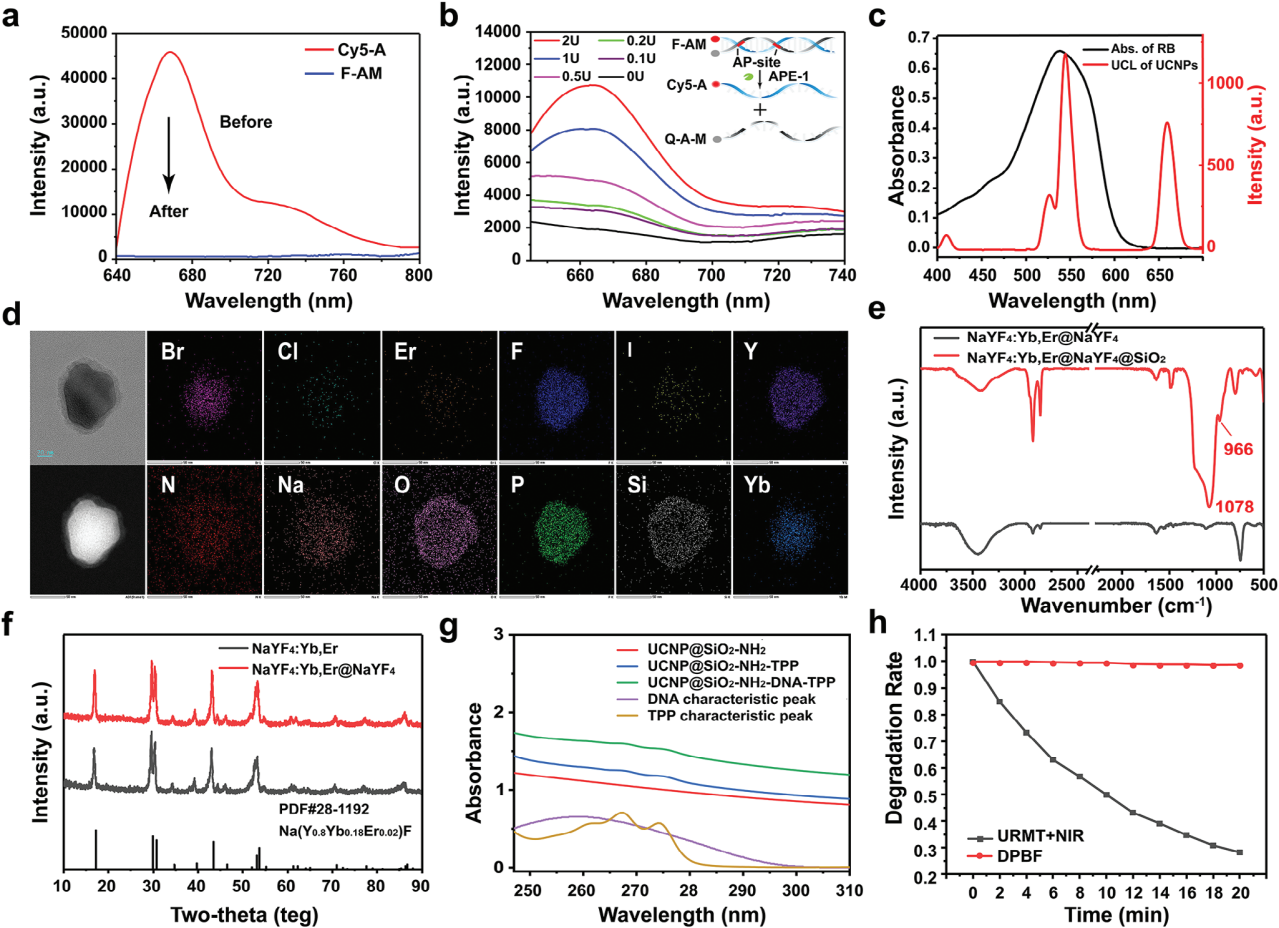
Characterizations of the URMT nanosystem. a) Fluorescence spectra of Cy5‐A (100 nM) before and after the formation of F‐AM. b) Fluorescence spectra of F‐AM (100 nM) with increased concentration of APE1. *Inset*: schematic illustration of the APE1‐mediated release of Cy5‐A from F‐AM. c) 980 nm near‐infrared laser excited UCL spectra of UCNPs and ultraviolet (UV)–visible (Vis) absorption spectrum of RB PCs. d) High‐angle annular dark‐field scanning transmission electron microscopy image and corresponding elemental mapping images of URMT. e) FITR of NaYF_4_:Yb,Er@NaYF_4_ and NaYF_4_:Yb,Er@NaYF_4_@SiO_2_. f) XRD patterns of NaYF_4_:Yb,Er and NaYF_4_:Yb,Er@NaYF_4_, and standard crystal diffraction peaks of Na(Y_0.8_Yb_0.18_Er_0.02_)F. g) UV/Vis absorption spectra of UCNP@SiO_2_‐NH_2_, UCNP@SiO_2_‐NH_2_‐TPP, UCNP@SiO_2_‐NH_2_‐DNA‐TPP, and characteristic peaks of DNA and TPP. h) ^1^O_2_ generation of URMT with 980 nm light irradiation determined by the time‐course of the degradation rate of DPBF.

The APE1‐triggered activation of URMT was evaluated through the mechanism of the Förster resonance energy transfer (FRET). Briefly, miR21 (labeled with a black hole quencher‐3) was mixed with Cy5‐modified anti‐miR21. The fluorescence intensity of Cy5 decreased significantly, suggesting the successful synthesis of the AM complex (labeled as F‐AM) (**Figure** **2**a). When APE1 was added, it can damage the phosphodiester bond at the site of AP, resulting in the release of Cy5‐A for an increased Cy5 signal (Figure 2b). The fluorescence had a proportional relationship with APE1 concentration. Additionally, time‐dependent fluorescence profiles indicated the rapid response of F‐AM to APE1‐triggered DNA cleavage (Figure S2, Supporting Information). In order to validate the APE1‐activated mechanism, nAM was constructed as a control group, which has an identical sequence with AM but lacking AP sites. Figure S3 (Supporting Information) showed that there was minimal fluorescence change for nAM, confirming the crucial role of the AP sites. Gel electrophoresis analysis further confirmed APE1‐triggered activation of AM (Figure S4, Supporting Information).

UCNPs, serving as both delivery tools and NIR‐to‐UV transducers, were synthesized via a thermal‐decomposition approach. Herein, oleate‐capped NaYF_4_:Yb,Er@NaYF_4_ core–shell UCNPs were synthesized. Yb/Er codoping in the core of UCNPs allowed for green emission under 980 nm excitation (Figure S5, Supporting Information), matching the maximum absorption of the sensitizer Rose Bengal (RB) (Figure 2c). The size of the core–shell structured UCNPs was ≈45 nm, and the shell thickness was ≈ 5 nm (Figure S6, Supporting Information). Transmission electron microscopy (TEM) images confirmed the monodispersity of UCNPs (Figure S7, Supporting Information). The UCNPs were then coated with mesoporous silica for loading RB on the pores to obtain UCNPs@mSiO_2_‐RB (UR). Subsequently, TPP was conjugated utilizing 1‐ethyl‐3‐diaminopropyl carbodiimide and *N*‐hydroxysuccinimide to form UCNPs@mSiO_2_‐RB‐TPP (URT). Finally, the AM were attached to the URT surface to form UCNPs@mSiO_2_‐RB‐TPP‐DNA (URMT). The constructed URMT was further characterized to by scanning electron microscope (SEM), corresponding elemental mapping images and energy dispersive spectrometer (EDS) scanning (Figure 2d and Figure S8, Supporting Information). The loading efficiency of RB and anti‐miR21 on URMT was calculated as 52.9% and 80.5%, respectively. The conjugation efficiency of TPP was calculated to be 59.8%. The composition and mass fraction of each element in URMT is listed in Table S1 (Supporting Information). The size of the constructed URMT was 108 nm, and the shell thickness was 8 nm (Figure S3, Supporting Information). Zeta potential results showed a positive charge of +8.2 mV for UCNPs@mSiO_2_@TPP, which decreased to −2.8 mV after the DNA functionalization (Figure S9, Supporting Information). In addition, URMT can maintain good stability in HEPES, PBS, and DMEM (Figures S10 and S11, Supporting Information). The crystalline phases and chemical structures of the synthesized core–shell UCNPs were analyzed using Fourier transform infrared (FTIR) spectroscopy and X‐ray diffraction (XRD) analysis. In the FTIR spectrum of NaYF_4_:Yb,Er@NaYF_4_ (Figure 2e), the band at 3421 cm^−1^ was due to the stretching vibration in hydroxyl groups of oleic acid modified on the surface of UCNPs. The peaks at 2854 and 2923 cm^−1^ were corresponded to the symmetric and asymmetric stretching vibrations of the methylene group, respectively. The red curve represents the FTIR spectrum after silica coating; three new peaks can be observed at 966 and 1078 cm^−1^. Additionally, XRD results confirmed nanoparticle formation. Figure 2f presented the XRD pattern of UCNPs, where all the marked diffraction peaks correspond to the β‐NaYF_4_ crystals. These results collectively indicated the successful construction of URMT.

After the successful synthesis of URMT, UV–visible (Vis)–NIR absorption measurements (Figure 2g and Figure S12, Supporting Information) showing characteristic peaks at ≈260 nm for DNA and a broad absorbance range (265–275 nm) for TPP confirmed the successful encapsulation of DNA and TPP in UR. Furthermore, the emission spectrum of UCNPs matched the absorbance spectrum of RB, indicating that RB can be effectively activated by UCNPs (Figure 2c). To assess singlet oxygen (^1^O_2_) generation, 1,3‐diphenylisobenzofuran (DPBF) photo‐oxidation analysis was performed using methanol. DPBF is a specific scavenger for ^1^O_2_, which forms an endoperoxide and decomposes into 1,2‐dibenzoylbenzene.^[^ ^61^ ^]^ UV–Vis spectra of URMT were recorded at different exposure times under 980 nm light irradiation. The absorption intensity at 415 nm exhibited a time‐dependent decrease, indicating DPBF decomposition because ^1^O_2_ was photogenerated by URMT with irradiation (Figure 2h). Importantly, no changes in absorption were observed at 415 nm without 980 nm light irradiation (Figure S13, Supporting Information).

### 2.2 Controlled Activation of URMT In Vitro

**Figure 3:**
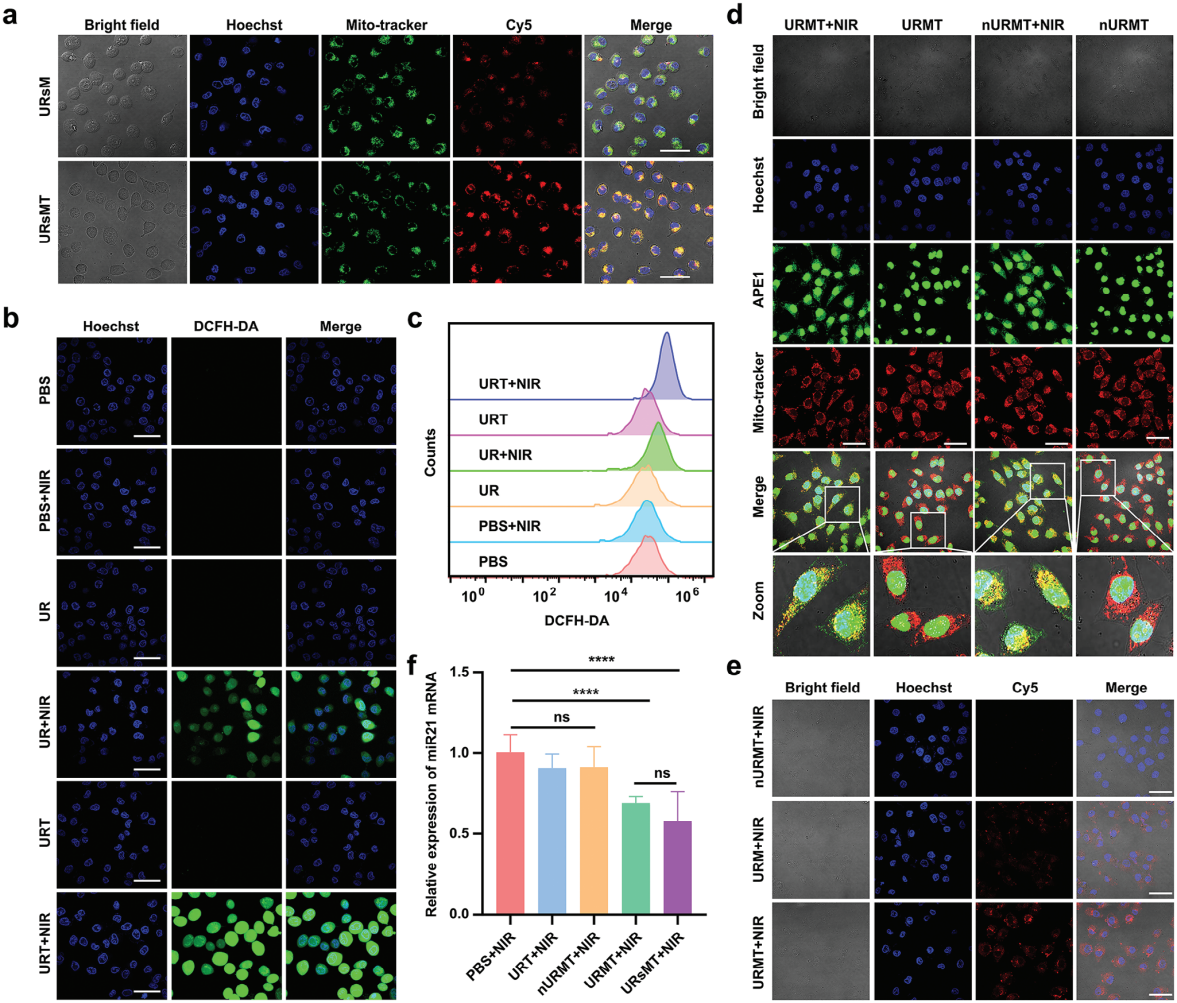
Controlled activation of URMT in MCF‐7 cells. a) CLSM images of MCF‐7 cells treated with URsM and URsMT (anti‐miR21 was labeled with Cy5) and then colocalized with MitoTracker (green) and Hoechst 33342 (blue). Scale bar, 50 µm. b) Fluorescence images staining with DCFH‐DA (green) and Hoechst 33342 (blue) showed intracellular ROS of MCF‐7 cells with different treatments. Scale bar, 50 µm. c) Flow cytometry analysis of intracellular ROS of MCF‐7 cells with different treatments. d) Immunofluorescence staining of APE1 in MCF‐7 cells treated with different materials with and without NIR light irradiation. Nuclei were stained blue, APE1 were stained green and MitoTracker were stained red. Scale bar, 50 µm. e) CLSM images of MCF‐7 cells treated with FRET pair‐labeled nURMT, URM, and URMT with or without 980 nm NIR light irradiation. Scale bar, 50 µm. f) qRT‐PCR analysis of the mRNA expression of miR21 in MCF‐7 cells upon different treatments. ****P < 0.0001. Data are presented as mean ±SD (*n* = 3).

The intracellular uptake efficiency and subcellular localization of URsM and URsMT (where sM represents the Cy5‐labeled single‐stranded anti‐miR21) were investigated using confocal laser scanning microscopy (CLSM) and flow cytometry. As illustrated in Figure S14 (Supporting Information), compared with URsM‐treated cells, the CLSM images of URsMT‐treated cells exhibited a stronger fluorescence intensity. Flow cytometry analysis further confirmed this result (Figure S15, Supporting Information). The colocalization study revealed that URsMT accumulated within the mitochondria of MCF‐7 cells, confirming that TPP has strong ability to mediate mitochondrial targeting (**Figure** **3**a). Additionally, the potential endocytosis mechanism of URsMT was further investigated using some selective inhibitors (Figure S16, Supporting Information). Chlorpromazine hydrochloride, a kind of inhibitor for clathrin‐mediated endocytosis can reduce cellular uptake less than 75%. Furthermore, 5‐(*N*‐ethyl‐*N*‐isopropyl) amiloride, a kind of inhibitor for micropinocytosis, inhibited ≈15% of the cellular uptake of URsMT. These results indicated that the main mechanisms underlying the cellular uptake of URsMT were clathrin‐mediated endocytosis and macropinocytosis. Subsequently, we assessed URT‐induced intracellular ROS production by using 2,7‐dichlorofluorescein‐diacetate (DCFH‐DA). URT‐treated MCF‐7 cells without irradiation showed weak fluorescence (Figure 3b,c and Figure S17, Supporting Information). In contrast, MCF‐7 cells displayed bright fluorescence after NIR irradiation. Furthermore, URT‐treated cells exhibited higher fluorescence intensity compared with UR‐treated cells with NIR irradiation. Following the determination of the photodynamic effect of URT, the controlled activation of URMT in vitro was investigated. Immunofluorescence analysis revealed that the mitochondrial APE1 level markedly increased in the nURMT + NIR and URMT + NIR groups compared with the nonirradiation groups (Figure 3d and Figure S18, Supporting Information). These results indicated that PDT stimulated APE1 translocation from the nucleus to mitochondria. Furthermore, the fluorescence intensity of URMT + NIR group was notably stronger than that of URM + NIR group and nURMT + NIR group (Figure 3e), suggesting that APE1 migration from the nucleus to mitochondria facilitated the cleavage of the targeted double‐stranded DNA (F‐AM) that was delivered to the mitochondria, thereby enabling the fluorescence recovery of anti‐miR21 (Cy5‐labeled single‐stranded anti‐miR21).

Studies have shown that miR‐21 plays an anti‐apoptotic role in various human malignancies.^[^ ^27^ ^]^ Overexpression of miR‐21 directly targets Bcl‐2, upregulating its expression and subsequently inhibiting apoptosis.^[^ ^62^ ^]^ Real‐time fluorescence quantitative polymerase chain reaction (qRT‐PCR) was performed to assess miR21 expression in the cytoplasm. The expression of miR21 markedly decreased in URMT‐treated MCF‐7 cells with irradiation, which was not significantly different from URsMT (loaded with a single strand of anti‐miR21) (Figure 3f). Conversely, nURMT‐treated cells (miR21 strand without the AP sites) exhibited no effect on miR21 expression. Moreover, URMT‐treated cells without NIR irradiation presented no change in miR21 expression, confirming that the enzymatic cleavage of the AP sites regulated miR21 expression (Figure S19, Supporting Information). The feasibility for enzyme‐activated miR21 regulation by NIR light control was further validated in different tumor cells (Figure S20, Supporting Information).

### 2.3 In Vitro Cytotoxicity

**Figure 4:**
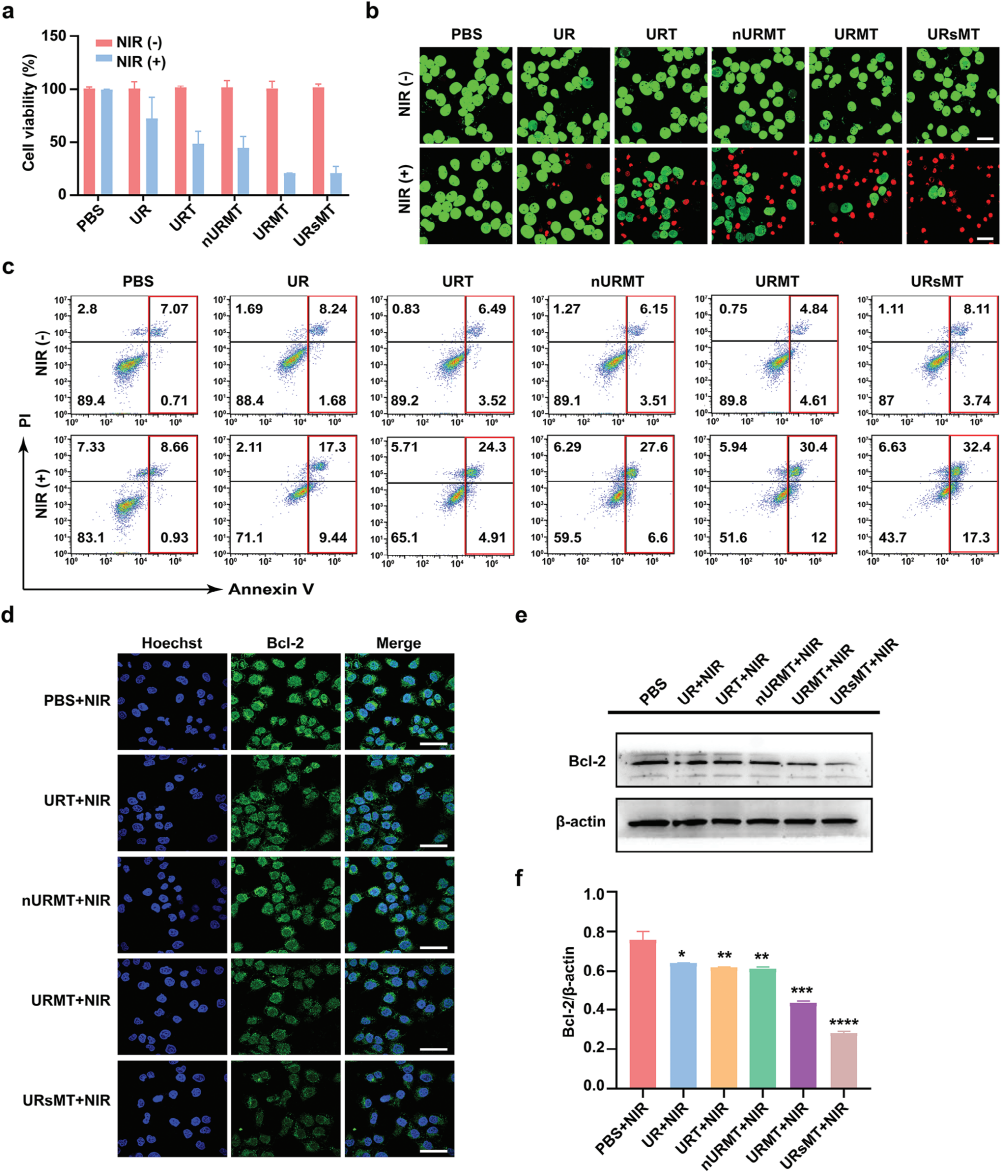
In vitro killing effects of nanosystem on MCF‐7 cells. a) Cell viability of MCF‐7 cells incubated with different treatments in the dark or with 980 nm laser irradiation. Data are presented as the mean ± SD (*n* = 3). b) Representative confocal fluorescence images of Calcein‐AM (green) and PI (red) co‐stained MCF‐7 cells exposed to different samples with and without NIR light irradiation. Scale bar, 50 µm. c) Cell apoptosis was analyzed by flow cytometry with Annexin V/PI double staining. d) Immunofluorescence staining of Bcl‐2 in MCF‐7 cells treated with different nanomaterials with NIR light irradiation. Nuclei were stained blue and Bcl‐2 was stained green. Scale bar, 50 µm. (e) Western blotting assay of the expression and f) quantification of Bcl‐2 protein in MCF‐7 cells with different treatments. Data are presented as the mean ± SD (*n* = 3). *P < 0.05, **P < 0.005, ***P < 0.001, ****P < 0.0001.

The in vitro antitumor efficiency of URMT was tested using a Cell Counting Kit‐8 (CCK8) assay (**Figure** **4**a). The nanomaterials showed good biocompatibility without NIR irradiation. However, under 980 nm light irradiation, the viability of cells treated with UR, URT, nURMT, URMT, and URsMT decreased to 70%, 47%, 43%, 20%, and 21%, respectively. The combined antitumor activity of URMT in MCF‐7 cells was further verified by Calcein/AM double staining assay and an Annexin V‐FITC/PI apoptosis detection assay. The results showed that URMT treatment following NIR light irradiation exhibited more red fluorescence signal for dead cells (Figure 4b and Figure S21, Supporting Information) and highest percentage of apoptosis (Figure 4c and Figure S22, Supporting Information). Notably, URMT exhibited no cytotoxicity to the normal cells (Figure S23, Supporting Information), further confirming its significant biocompatibility in vitro.

The mitochondria membrane potential (MMP) was determined using the JC‐1 fluorescent probe. The decrease in MMP caused the color change of the probe from red to green during apoptosis.^[^ ^63^ ^]^ As shown in Figure S24 (Supporting Information), URMT‐treated MCF‐7 cells with irradiation exhibited a stronger green signal and a weaker red signal. This finding suggests that URMT can damage mitochondria and decrease MMP. In this study, Western blot and immunofluorescence analysis assay showed a significant decrease in Bcl‐2 levels in MCF‐7 cells after URMT treatment with NIR irradiation (Figure 4d,e). These findings together indicated that URMT can induce cell apoptosis by a mitochondria‐involved apoptosis pathway.

### 2.4 Tumor‐Targeting and Biodistribution of URMT In Vivo

**Figure 5:**
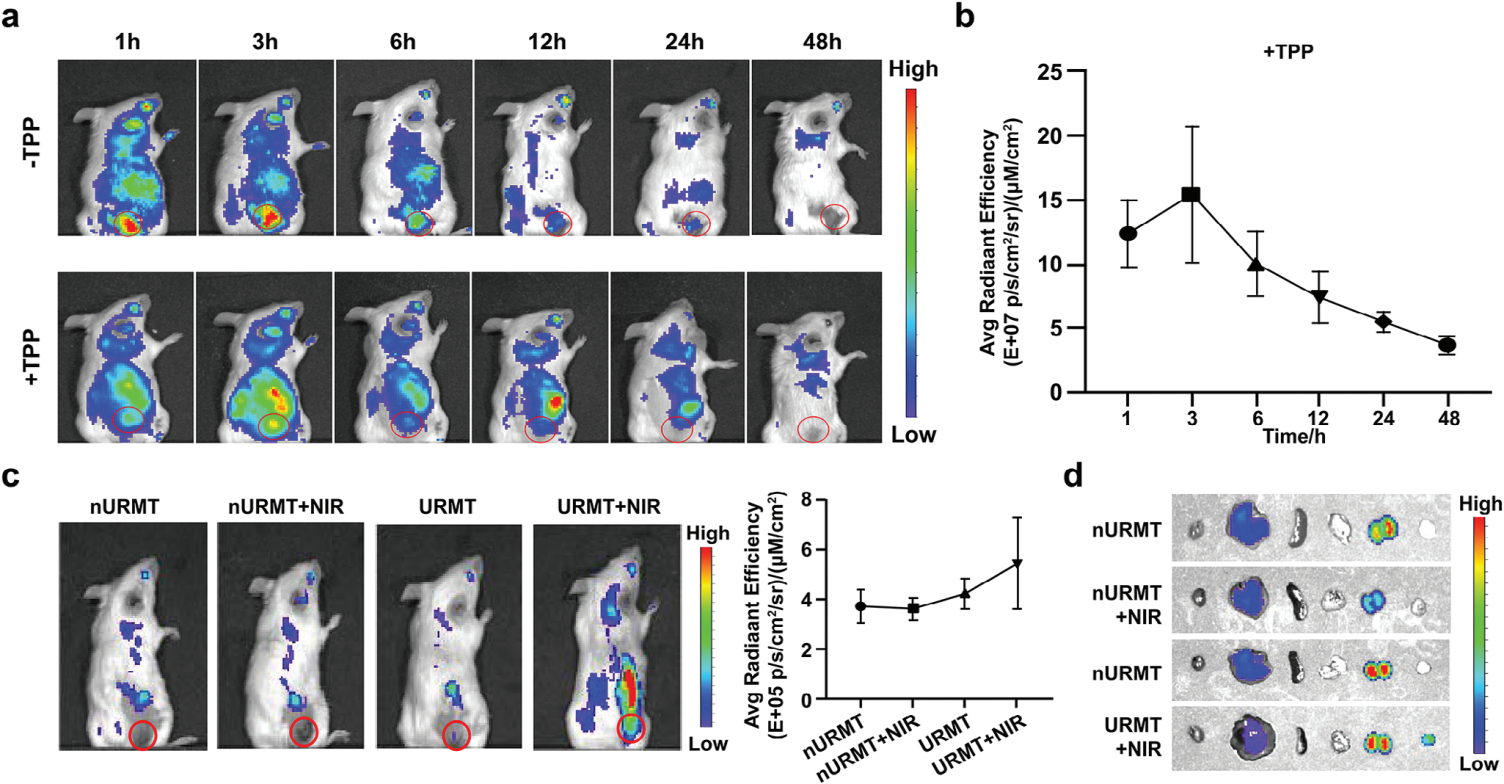
In vivo fluorescence imaging of the URMT nanosystem. a) Representative whole‐body fluorescence images of 4T1 tumor‐bearing mice injected intravenously with URsM and URsMT at the tumor site. Tumors are indicated by red circles. b) Quantitative analysis of fluorescence intensities at tumor sites in panel. Data are presented as the mean ± SD (*n* = 4). c) Real‐time fluorescence imaging of representative tumor‐bearing mice after i.v. injection of FRET pair‐labeled nURMT or URMT followed with or without NIR light irradiation. Tumors were indicated with red circles (left). Quantitative analysis (right) of fluorescence intensities at tumor sites. Data are presented as mean ± SD (*n* = 4). d) Ex vivo fluorescence images of major organs and tumors from mice after different treatments. From left to right: heart, liver, spleen, lung, kidney, and tumor.

Nanoparticles usually have high permeability and retention effects.^[^ ^64^, ^65^ ^]^ Pharmacokinetic analysis showed that URMT nanoparticles maintained higher blood concentrations in vivo for 12 h compared with RB (Figure S25, Supporting Information). Furthermore, we evaluated the time‐dependent biodistribution of URMT in tumor‐bearing mice. URsM and URsMT (Cy5‐labeled anti‐miR21) were administrated to the mice via tail vein injection. The organs and tumors of the mice at different times postinjection were imaged. As shown in **Figure** **5**a, the Cy5 signal at the tumor site showed a significant increase, reaching a peak at 3 h and then decreasing from 6 to 48 h. Quantitative analysis indicated that the fluorescence intensity of URsMT treatment at 3 h was ≈ 1.2‐, 1.2‐, 1.7‐, 2.2‐, and 3.3‐ fold higher compared with those at 1, 6, 12, 24, and 48 h, respectively (Figure 5b). Additionally, the Cy5 fluorescence intensity of the tumor site in URsMT treatment was significantly stronger than that in URsM treatment, suggesting that the URsMT can target the tumor site. As shown in Figure S26 (Supporting Information), the fluorescence intensity of anti‐miR21 in organs was highly dependent on the time, mainly accumulated in liver and cleared through renal metabolism.

To validate the in vivo NIR‐triggered activation, tumor‐bearing mice were intravenously injected with FRET pair‐labeled URMT and nURMT. NIR irradiation was applied to the tumor site at 3 h postinjection, a time point when the tumor showed the highest material accumulation. Notably, URMT showed stronger fluorescence intensity compared with nURMT (Figure 5c). The intratumoral fluorescence signal was increased in URMT with irradiation group, which was due to the release of anti‐miR21 by the NIR‐induced APE1 translocation. Additionally, the ex vivo imaging results of tumors and normal organs were consistent with the in vivo imaging results (Figure 5d).

### 2.5 In Vivo Antitumor Therapy

**Figure 6:**
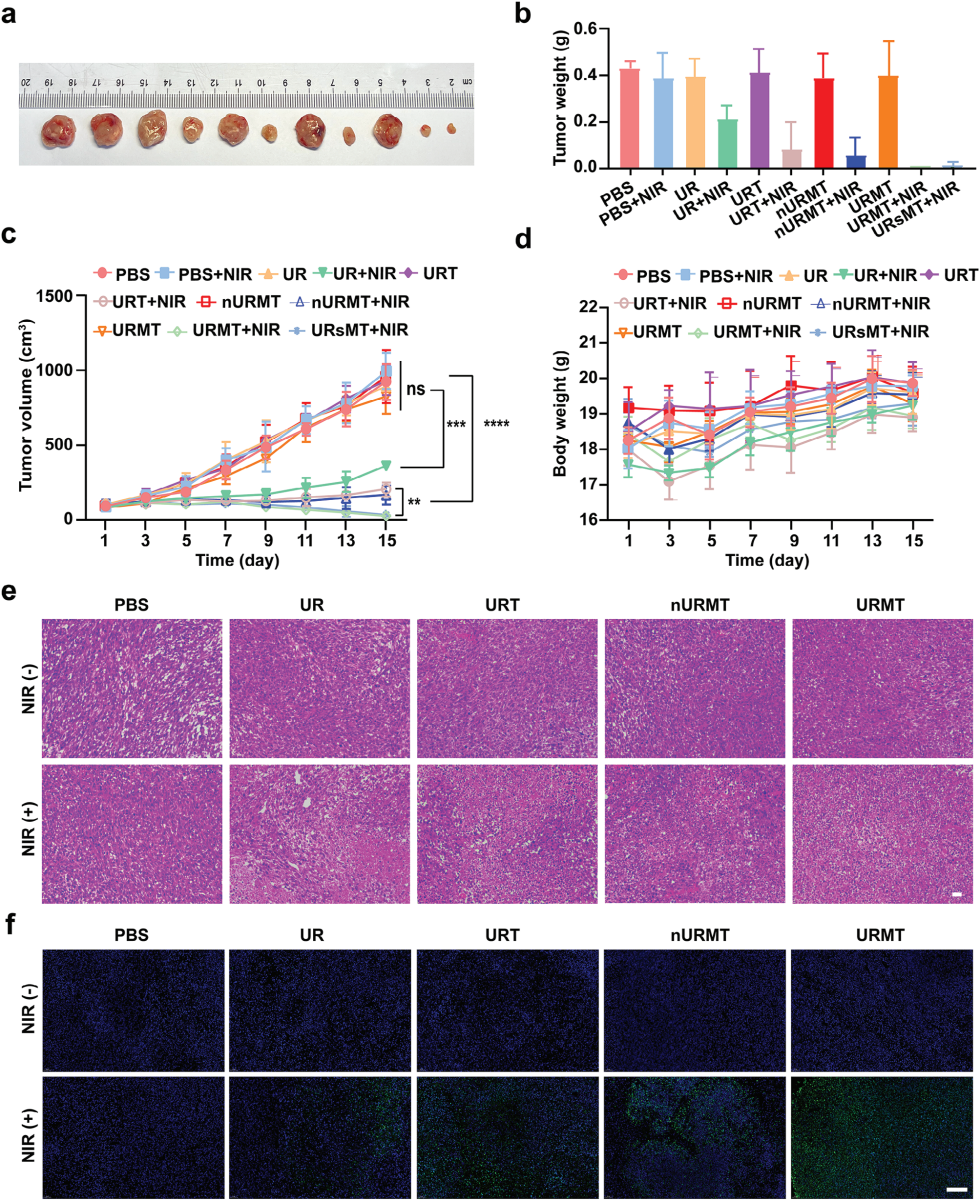
In vivo synergistic antitumor therapy of nanosystem. a) Representative photographs of the excised tumor with different treatments. b) Tumor weight in the different treatment groups. Data are presented as mean ± SD (*n* = 5). c) Tumor growth curves in the different treatment groups at different times. Data are presented as means ± SD (*n* = 5). **P < 0.005, ***P < 0.001, ****P < 0.0001. d) Body weight of 4T1 tumor‐bearing mice in the different treatments. Data are presented as mean ± SD (*n* = 5). e) Hematoxylin and eosin (H&E) and f) terminal deoxynucleotidyl transferase‐mediated deoxyuridine triphosphate nick end labeling (TUNEL) staining of the tumor sections with different treatments. Scale bar, 100 µm.

Based on the remarkable performance of the as‐prepared nanomaterials in vitro, we performed an in‐depth evaluation of the in vivo antitumor efficacy and safety of URMT. BALB/c mice bearing 4T1 tumor xenografts were intravenously administered with PBS, UR, URT, URMT, nURMT, and URsMT thrice every other day. In the NIR irradiation groups, the tumor site was exposed to 980 nm laser irradiation for 10 min at 3 h postinjection. The tumor size or weight in PBS, PBS + NIR, UR, and URT groups showed no significant difference (**Figure** **6**a–c), suggesting that 980 nm laser irradiation, UR or URT alone cannot affect the tumor growth. When treated with URMT + NIR, the tumor growth was effectively inhibited, while there was no inhibition effect in the tumor progression of URMT, indicating 980 nm laser‐induced results. Furthermore, nURMT treatment exhibited no antitumor effect. The hematoxylin and eosin (H&E) staining and TUNEL staining of tumor sections showed that the URMT + NIR group induced significant necrosis and apoptosis in tumor tissue (Figure 6e,f). Moreover, there was no significant difference between the URMT + NIR group and the URsMT group in inducing tumor necrosis and apoptosis (Figure S27, Supporting Information). Additionally, there was no significant difference in weight change among the ten groups (Figure 6d).

The controlled activation of URMT in vivo was further investigated. As shown in Figure S28 (Supporting Information), APE1 was predominantly localized in the nucleus of tumor tissue without 980 nm light irradiation. After 980 nm light irradiation, a portion of APE1 migrated out of the nucleus. These findings demonstrate that our nanosystem can achieve the migration of APE1 from the nucleus to the cytoplasm in vivo. As shown in Figure S29 (Supporting Information), the expression of miR21 markedly decreased in URMT‐treated mice with 980 nm light irradiation. Conversely, nURMT‐treated cells exhibited no effect on miR21 expression compared with that in the control group. The results in vivo were consistent with those observed at the cellular level. Immunofluorescence analysis showed a significant decrease in Bcl‐2 level in tumor after URMT treatment with NIR irradiation (Figure S28, Supporting Information).

To further determine the safety of URMT, we analyzed various indicators. As shown in Figure S30 (Supporting Information), no obvious difference of biochemical parameters was found in the serum levels of glutamic‐pyruvic transaminase (ALT), glutamic oxalacetic transaminase (AST), alkaline phosphatase (ALP), blood urea nitrogen (BUN), creatinine (CREA), total cholesterol (TCHO), triglyceride(TG), total protein (TP), and blood glucose (GLU). The cytokines IL‐12 and IL‐6 in serum, used to access systematic inflammation, did not show significant changes (Figure S31, Supporting Information). Furthermore, complete blood was collected for hematological parameters, and all groups were within the normal range (Figure S32, Supporting Information). Ultimately, major normal organs (heart, liver, spleen, lung, and kidney) were collected and sectioned for histological analysis, and H&E staining results showed no distinct damage in any of the groups (Figure S33, Supporting Information), further confirming the low systemic toxicity of URMT and its promising potential for subsequent clinical applications.

To ensure consistency between in vitro and in vivo experiments, a tumor treatment experiment was conducted using BALB/c nude mice inoculated with MCF‐7 cells. URMT was administered via tail vein injection, and the tumor site was irradiated with a 980 nm laser (1.2 W cm^−2^, 10 min) at 3 h postinjection. The results showed that URMT treatment with NIR light irradiation significantly inhibited tumor growth, while the URMT group without light irradiation showed no significant difference compared with the PBS group. This further demonstrated the feasibility of the system we designed. (Figure S34, Supporting Information).

## 3 Conclusion

To conclude, in this study, we successfully constructed a NIR light‐mediated, UCNP‐based hybrid nanosystem for combinational tumor therapy. We used NIR light as an inducer for PDT to initiate enzymatically regulated gene expression. This nanosystem showed good efficacy both in vitro and in vivo. The UCNPs acted as a light transducer, allowing NIR light‐induced generation of ROS in the mitochondria. This promotes the subcellular translocation of APE1 and partially damages the mitochondrial membrane. APE1 cleaves the AP site of the rationally designed oligonucleotide, which released functional oligonucleotide complementary strands into the cytoplasm to bind small RNAs in the cytoplasm, thereby initiating apoptosis. By combining NIR light‐induced mitochondrial photodamage with activatable gene regulation, this nanosystem achieves accurate spatial and temporal control for combinational tumor treatment. This approach can considerably expand the techniques available for developing high‐precision combinational tumor therapies.

## 4 Experimental Section

### Materials and Reagents

Rare earth oxides, oleic acid (OA, 90%), 1‐octadecene (ODE, 95%), and oleylamine (OM, 90%) were obtained from Acros. Trifluoroacetic acid (99%), APTES, TEOS, and Sulfo‐SMCC were purchased from Sigma‐Aldrich. DCFH‐DA, Hoechst 33342, ROS Assay Kit and Mitochondrial Membrane Potential Assay Kit with JC‐1 obtained from Solarbio (Beijing, China). Calcein/AM double staining kit and Mito‐Tracker Green/Red CMXRos obtained from Beyotime (Beijing, China). Annexin V/PI apoptosis kit was purchased from Elabscience (Wuhan, China). Cell Counting Kit‐8 (CCK‐8) was bought from NCM Biotech (Suzhou, China). All HPLC‐purified DNA molecules were synthesized by Sangon Biotech Co., Ltd. (Shanghai, China). Dulbecco's modified Eagle's medium (DMEM), RPMI 1640 medium, fetal bovine serum (FBS), phosphate buffered saline (PBS), trypsin and penicillin‐streptomycin were obtained from Wisent Bio Co., Ltd. (Nanjing, China). Opti‐MEM was purchased from Gibco (California, USA). Matrigel was purchased from Corning (USA). 35‐mm glass bottom dishes (Cellvis) were purchased from Beijing Haimabor Biotechnology Co., Ltd. (Beijing, China). The water used throughout the experiments was Millipore water (18.2 MΩ). All the chemicals were used as received without further purification.

### Instruments

The transmission electron microscopic (TEM) images were captured on the JEM‐1200EX transmission electron microscope (JEOL, Japan). The scanning electron microscope (SEM) images were obtained from the SU8220 scan electron microscope (Hitachi Co. Ltd, Japan). Fluorescence spectra were captured on an Edinburgh FS5C fluorimeter (UK). UV–Vis spectra was recorded by an Edinburgh DS5 spectrophotometer (UK). Cell viability data were obtained using a Thermo MULTISCAN GO reader (Thermo, USA). The CLSM images were obtained using an Olympus FluoView FV1000 confocal microscope (Olympus Corporation, Japan). The flow cytometry assays were carried out using a Guava easyCyte flow cytometer 6 (Guava Technologies Inc., USA). The in vivo fluorescence images were obtained on an IVIS® Spectrum in vivo imaging system (PerkinElmer Inc., USA).

### Synthesis of NaYF_4_:Yb,Er UCNPs

A mixture containing 5.64 g OA, 5.04 g ODE, 0.78 mmol YCl_3_, 0.2 mmol YbCl_3_, and 0.02 mmol ErCl_3_ was added to a three‐necked flask (100 mL) at room temperature. The solution was gradually heated to 120 °C and continuously stirred for 1 h. Upon cooling to room temperature, NaOH (2.5 mmol) and NH_4_F (4 mmol) were dissolved in 2 mL methanol before slowly adding into mixture. The temperature was then raised to 90 °C and maintained for 30 min. After the methanol is removed, the temperature was raised to 120 °C for 35 min. The system was then heated to 330 °C in nitrogen atmosphere for 90 min. After cooling, the precipitation was obtained by adding ethanol, and then dissolved in 10 mL cyclohexane by centrifugal collection.

### Synthesis of Trifluoroacetate Coated Core–Shell Structured NaYF_4_:Yb,Er@NaYF_4_ UCNPs

The prepared NaYF_4_:Yb,Er (5 mL) colloidal solution was added to the solution containing the solvent (5.64 g OA and 5.04 g ODE) and a mixture of shell precursors (0.5 mmol CF_3_COONa and 0.5 mmol Y(CF_3_COO)_3_). After being heated to 120 °C for 40 min under vacuum, the mixture was heated to 315 °C under nitrogen protection for 60 min. After cooling, the precipitation was obtained by adding ethanol and collected by centrifugation.

### Synthesis of URMT

First, 0.1 g CATB was mixed with 20 mL deionized water and 1 mL of UCNPs, and the mixture was stirred overnight. Next, 40 mL deionized water, 6 mL of ethanol, and 100 µL of 2 mol sodium hydroxide were added to the mixture, and the temperature was raised to 60 °C. An ethanol solution of TEOS (200 µL TEOS, 1 mL ethanol) was then added, and the reaction was stirred for 1 h. Dropwise addition of APTES (100 µL APTES, 1 mL ethanol) followed, and the solution was heat and stirred for 30–60 min. The resulting precipitation (UCNPs@mSiO_2_) was collected by centrifugation and then dispersed in 2 mL deionized water. 1 mL of UCNPs@mSiO_2_ and 1 mL RB (1 mg/mL) were mixed and stirred in the dark for 24 h. Then the precipitation (UCNPs@mSiO_2_‐RB, UR) was collected by centrifugation and dispersed in 1 mL deionized water. In a separate step, a mixture of 2 mg TPP‐COOH, 2.4 mg NHS, 7.2 mg EDC, 480 µL methanol, and 120 µL sodium carbonate buffer (0.05 M, pH 9.5) was stirred for 30 min. After the reaction, 1 mL of UR was added, collected by centrifugation and redispersed into 1 mL of water to obtain UCNPs@mSiO_2_‐RB‐TPP (URT). Furthermore, a 100 mM anti‐miR21 chain and its complementary chain mother liquor were prepared with water. A total of 10 µL of anti‐miR21, 10 µL of complementary chain, and 30 µL of HEPES buffer (20 mM) were mixed and heated in a metal bath at 95 °C for 5 min. The mixture was then annealed on ice to room temperature, followed by incubation in the dark for 12 h to ensure adequate hybridization. Finally, 20 µL of the above dsDNA solution was added into 50 µL of URT, vortexed for 5 min and stood for 1 h at room temperature in the dark.

### Singlet Oxygen Detection

The DMSO solution of DPBF (1 mg/mL) was added to a cuvette containing UR. Then, the corresponding absorption spectra were measured under dark conditions or at different irradiation time with 980 nm NIR laser irradiation (1.2 W cm^−2^).

### APE1 Induced Activation Detection

The F‐AM probes were generated by hybridization of miR21 labeled with a BHQ3 quencher and AP site (Q‐A‐M) with the Cy5‐modified anti‐miR21 (Cy5‐A). Briefly, stock DNAs of Q‐A‐M strand and Cy5‐A strand (100 µM in Millipore water) were diluted to 20 µM in HEPES buffer. The mixture was heated (95 °C, 5 min) and then cooled to room temperature. The F‐AM probes (100 nM) was incubated with different concentration (0.1, 0.5, 1, and 2 U mL^−1^) of APE1 in Tris buffer (20 mM, pH 7.4) at 37 °C for 30 min. Moreover, the F‐AM probes (100 nM) was incubated with a given concentration of APE1 in Tris buffer at 37 °C for different times. The emission spectra were recorded by a fluorescence spectrophotometer.

### Gel Electrophoresis Analysis of Oligonucleotide Damage

The F‐AM probes and APE1 was incubated in the reaction buffer at 37 °C for 30 min. Then, the F‐AM probes were collected by centrifugation and analyzed by electrophoresis using nucleic acid nondenatured polyacrylamide gel preparation kit (Solarbio).

### Stability of URMT

The stability of URMT was determined by detecting DNA fluorescence recovery of URMT at different time points (0, 1, 2, 4, 6, 8 h) in different buffers (20 mM HEPES, PBS, and DMEM). The supernatant of URMT was centrifuged at 13 000 × *g* for 10 min at 4 °C and collected at different time points to detect the fluorescence changes. Moreover, the stability of URMT was determined by detecting the change of DLS at different time points in different buffers (20 mM HEPES, PBS, H_2_O, and DMEM).

### Cell Culture

MCF‐7 cells, 4T1 cells, 16HBE cells, A549 cells, B16F10 cells, and HeLa cells were cultured in an incubator (5% CO_2_, 37 °C). The medium used for MCF‐7 cells and Hela cells was DMEM supplemented with 10%FBS and 1% penicillin‐streptomycin. The medium used for 4T1, A549 cells, B16F10 cells and 16HBE cells was RPMI 1640 supplemented with FBS (10%) and penicillin‐streptomycin (1%).

### Cellular Uptake of URMT

MCF‐7 cells were seeded in 35 mm glass‐bottomed culture dishes or six‐well plates for 24 h. URsM and URsMT (DNA using a single strand anti‐miR21 labeled Cy5) were incubated with MCF‐7 cells in Opti‐MEM respectively. After 4 h, the cells were washed with PBS and fixed with 4% paraformaldehyde at 4 °C for 8 min. The cells were stained with Mito Tracker Green and Hoechst 33342 according to the instructions provided, and then visualized using CLSM. The cells in 6‐well plates were detached using trypsin and harvested for flow cytometric analysis. For NIR light‐activated experiments, MCF‐7 cells were incubated with nURMT, nURMT + NIR, URMT, and URMT + NIR for 4 h. Then, the light group cells were exposed to 980 nm laser (1.2 W cm^−2^, 1 min irradiation, and 5 min break) for 10 min. The cells were washed with PBS, fix with 4% paraformaldehyde and stained with Hoechst 33342, and then examined under CLSM imaging system.

### Cell Cytotoxicity Assay

To investigate the cell cytotoxicity of different groups, MCF‐7 cells were seeded in 96‐well plates and incubated for 24 h. The cells were treated with PBS, UR, URT, nURMT, URMT, and URsMT in Opti‐MEM for 4 h. After irradiated with 980 nm laser (1.2 W cm^−2^) for 10 min, the cells were incubated for another 24 h. Subsequently, 10 µL of CCK‐8 was added and incubated for 1 h. The absorbance of medium at 450 nm was measured by a microplate reader. To evaluate the cytotoxicity of nanosystem on normal cells, CCK‐8 assays were performed on 16HBE cells treated with different nanomaterials. After 24 h incubation, CCK‐8 assays were carried out following the same procedure as described above.

### Live/Dead Cell Analysis

MCF‐7 cells were seeded for 24 h. Subsequently, the cells were treated with PBS, UR, URT, nURMT, URMT, and URsMT for 4 h. Following the treatment, the cells were exposed to 980 nm NIR light (1.2 W cm^−2^) or for 10 min, and then incubated for 1 h. Subsequently, the cells were stained with the Calcein/PI Cell Viability/Cytotoxicity Assay Kit for 30 min in the dark, and imaging was performed by CLSM.

### Cell Apoptosis

MCF‐7 cells were seeded in 6‐well plates and incubated overnight. The medium was replaced, and PBS, UR, URT, nURMT, URMT, and URsMT were added for a 4 h treatment. Subsequently, the cells were irradiated with 980 nm NIR light (1.2 W cm^−2^) for 10 min, followed by an additional 1 h incubation. After the 1 h incubation, the cells were collected and washed with PBS. The cell suspension was incubated with Annexin V‐FITC and PI for 15 min. Finally, cell apoptosis was analyzed using a flow cytometer.

### ROS Analysis

MCF‐7 cells were cultured on glass bottom dishes or 6‐well plates for 24 h. Subsequently, the cells were treated with PBS, UR or URT for 4 h. Afterward, the cells were irradiated with 980 nm NIR light (1.2 W cm^−2^) for 10 min. Then, the cells were washed with serum‐free medium and incubated with DCFH‐DA (10 µM) for 20 min. Finally, the cells were stained with Hoechst 33 342 for 10 min and examined using CLSM imaging system. For flow cytometry analysis, the cells in 6‐well plates were detached using trypsin and then harvested.

### qRT‐PCR Analysis

MCF‐7 cells, A549 cells, B16F10 cells, and Hela cells were seeded in 6‐well plates for 24 h. The cells were then treated with PBS, URT, nURMT, URMT, and URsMT for 4 h. Subsequently, the cells were irradiated with 980 nm NIR light (1.2 W cm^−2^) for 10 min. After 36 h, total RNA was extracted using SanPrep Column microRNA Mini‐Preps Kit. The first strand cDNA was reversely transcribed from miR‐21 RT‐primer sequence using the PrimeScript II 1st strand cDNA Synthesis Kit. The qPCR reaction was performed using The PerfectStart Green qPCR SuperMix (Transgene Biotech) according to the manufacturer's instructions and was detected with the Applied Biosystems 7500 real‐time PCR detection system. Then, the expression levels were calculated following the standard ΔΔCt method. The primers used for real‐time PCR were list in Table S2 (Supporting Information).

### Western Blot

MCF‐7 cells were seeded in 6‐well plates for 24 h. The medium was then replaced with Opti‐MEM containing PBS, UR, URT, nURMT, URMT, and URsMT. After a 4 h incubation, the cells were irradiated with 980 nm laser (1.2 W cm^−2^) for 10 min. Then, the cells were washed with PBS, and added 1.5 mL of fresh DMEM medium. After 36 h, total proteins were extracted by lysed cells on ices with RIPA lysis buffer. The protein concentration was determined using a BCA Protein Assay Kit (Beyotime). The proteins were separated by 10% SDS‐PAGE gel and transferred onto a nitrocellulose filter (NC) membrane. The membrane was then blocked with 5% skim milk in TBS containing 0.25% Tween‐20 (TBST) for 1.5 h at room temperature. Primary antibodies against Bcl‐2 (1:1000, Immunoway) and β‐actin (1:1000, Immunoway) were used to probe the membrane at 4 °C overnight. Afterward, the membrane was incubated with HRP‐goat antirabbit/mouse IgG diluted 5000 times for 1 h. Finally, protein detection was performed using the enhanced chemiluminescence solution reaction.

### Immunofluorescence Staining

MCF‐7 cells were seeded in 35 mm glass‐bottomed culture dishes for 24 h. The cells were then treated with PBS, URT, nURMT, URMT, and URsMT for 4 h. After treatment, the cells were irradiated with 980 nm NIR laser (1.2 W/cm^−2^) for 10 min and incubated for 1 h. Subsequently, the cells were fixed with 4% paraformaldehyde, permeabilized with 0.1% Triton X‐100, and washed thrice with PBS. The cells were then blocked with 5% BSA for 1 h, followed by staining with primary antibody against Bcl‐2 (Immunoway, 1:500) and APE1 (Abcam, 1:500). Afterward, the cells were counterstained with Alexa Fluor 488 conjugated goat anti‐rabbit antibody (Abcam, 1:500). Finally, the cells were stained with Hoechst 33 342 and examined using CLSM imaging system.

### Analysis of Mitochondrial Membrane Potential

MCF‐7 cells were seeded in 35 mm glass‐bottomed culture dishes for 24 h. The cells were then treated with PBS, UR, URT, nURMT, URMT, and URsMT in Opti‐MEM for 4 h. Following treatment, the cells were irradiated with 980 nm NIR laser (1.2 W cm^−2^) for 10 min, and further incubated for 1 h. Subsequently, the cells were washed twice and JC‐1 probe was added for 20 min at 37 °C. Following two washes with JC‐1 staining buffer, the cells were stained with Hoechst 33342 and examined using CLSM imaging system.

### Mice and Tumor Models

The wild female BALB/c mice (16–18 g) and female BALB/c nude mice (16‐18 g) were brought from Beijing Vital River Laboratory Animal Technology Co., Ltd. The 4T1 tumor xenograft mouse model was established by subcutaneous injection of 4T1 cells (1×10^6^ cells with 100 µL, 1:1 (V/V) PBS and Matrigel). The MCF‐7 tumor‐bearing mice model was established by subcutaneous injection of MCF‐7 cells (1 × 10^6^ cells, 100 µL, 1:1 (V/V) PBS and Matrigel). The tumor volume (V) is calculated by the formula V = (length × width^2^)/2, where L and W represent the longest and shortest lengths, respectively. All in vivo experiments were carried out complying with NIH guidelines, and the experimental protocol was approved by the Institutional Animal Care and Use Committee of Beijing Tuberculosis and Thoracic Tumor Research Institute (NO. 2020‐013).

### In Vivo Imaging

Tumor‐bearing BALB/c mice were randomly divided into two groups (*n* = 5) to receive URsM and URsMT (Cy5‐labeled anti‐miR21 at a DNA dose of 25 nmol kg^−1^), respectively. The IVIS Imaging System was used to perform fluorescence imaging on various organs and tumors at different time points (1, 3, 6, 12, 24, and 48 h) after injection. Tumors and major organs were resected for imaging.

For the NIR light‐activated experiments, tumor‐bearing BALB/c mice were randomly divided into two groups (*n* = 4) to Cy5‐labeled URMT and nURMT, respectively. NIR light irradiation was conducted on the tumor site 1 h after injection using 980 nm laser (1.2 W cm^−2^) for 10 min. Mice were imaged at specified time intervals after irradiation using an IVIS Imaging System, and tumors and major organs were resected for in vitro imaging.

### Pharmacokinetics

48 BALB/c mice were randomly divided into 2 groups and were respectively injected with RB (1.6 mg k^−1^g) and URMT solution (containing RB 1.6 mg k^−1^g) through the tail vein for pharmacokinetic analysis. Then, the eyeball blood samples were collected from at 0.5, 1, 2, 4, 6, 8, 10, and 12 h, which were taken from three mice in each group at each time point (n = 3). Then, the plasma was collected by centrifugation at 3000 rpm for 10 min. The drug concentrations of RB, URMT nanoparticles were then extracted with acetonitrile and measured by fluorescence spectroscopy and high performance liquid chromatography (HPLC).

### Antitumor Activity Analysis in a 4T1 Tumor Model

When the tumor sizes reached ≈50 mm^3^, the tumor‐bearing BALB/c mice were randomly divided into eleven groups (n = 5) for the treatment with the following treatment: PBS, PBS + NIR, UR, UR + NIR, URT, URT + NIR, nURMT, nURMT + NIR, URMT, URMT + NIR, and URsMT + NIR. The mice were intravenously injected (i.v.) with a sample dose of 25 nmol kg^−1^. The injections were administrated thrice, with a frequency of once every 2 days. After 3 h injection, the tumor regions were exposed to 980 nm laser (1.2 W cm^−2^) for 10 min. Tumor size and body weight of each group were regularly monitored every 2 days. After 14 days, the animals were sacrificed, and the tumors were collected, weighted, and imaged. The isolated tumors were fixed in 4% paraformaldehyde and embedded in paraffin. Thick sections of the excised tumors were processed with H&E staining and TUNEL.

### Evaluating the Biosafety and Biocompatibility

Healthy BALB/c mice were intravenously injected with PBS, UR, URT, nURMT, and URMT (DNA: 25 nmol kg^−1^), respectively. After 14 days, the mice were sacrificed, and blood samples were collected. Completed blood counts were performed using the collected whole blood. The serum was obtained by centrifuging at 3000 rpm for 10 min at 4 °C. The supernatant of serum was used for biochemistry assessment using an automatic biochemical analyzer. Additionally, major organs including heart, liver, spleen, lung and kidney were harvested for standard H&E staining.

### In Vivo Treatment in MCF‐7 Tumor‐Bearing Mice Model

The MCF‐7 tumor‐bearing mice were randomly divided into four groups: PBS, PBS + NIR, URMT, and URMT + NIR. For the groups with laser irradiation, the tumor area was irradiated with 980 nm laser (1.2 W cm^−2^) for 10 min after 3 h injection. The tumor size and body weight were regularly monitored every 2 days. After 14 days, the animals were sacrificed, and the tumors were collected, weighted and imaged.

### Abbreviations

Abbreviations used in this paper are listed in Table S3 (Supporting Information).

### Statistical Analysis

All experiments were independently replicated at least three times. The data are presented as mean ± standard deviation (SD). Statistical analysis was performed using Student's *t*‐test and one‐way ANOVA. *P < 0.05, **P < 0.01, ***P < 0.001 and ****P < 0.0001 were considered as statistically significant. All data were analyzed using GraphPad Prism version 8 software.

## Conflict of Interest

The authors declare no conflict of interest.

## Supporting information

Supporting Information

## Data Availability

The data that support the findings of this study are available from the corresponding author upon reasonable request.
