# Supplementary material for: A Spatiotemporally Controlled Gene‐Regulation Strategy for Combined Tumor Therapy Based on Upconversion Hybrid Nanosystem
Source: Adv Sci (Weinh). 2024 Aug 29;11(40):2405640. doi: 10.1002/advs.202405640 (PMC11515897; doi:10.1002/advs.202405640)
Supplement: Supplementary file 1 — Supporting Information [file ADVS-11-2405640-s001.docx]

A Spatiotemporally-Controlled Gene-Regulation Strategy for Combined Tumor Therapy Based on Upconversion hybrid Nanosystem

Fang Wang^1^, Zechao Liu^2^, Yuechen Liu^2^, Jiayi Zhang^1^, Weizhe Xu^1^, Bei Liu^2^*, Zhaogang Sun^1^*, Hongqian Chu^1^*

1 Translational Medicine Center, Beijing Chest Hospital, Capital Medical University, 9 Beiguan Street, Beijing 101149, China
2 College of Science, Minzu University of China, 27 Zhongguancun South Avenue, Beijing 100081, China

*Corresponding author: sunzhaogang@bjxkyy.cn, chuhongqian@bjxkyy.cn.

Supporting Information


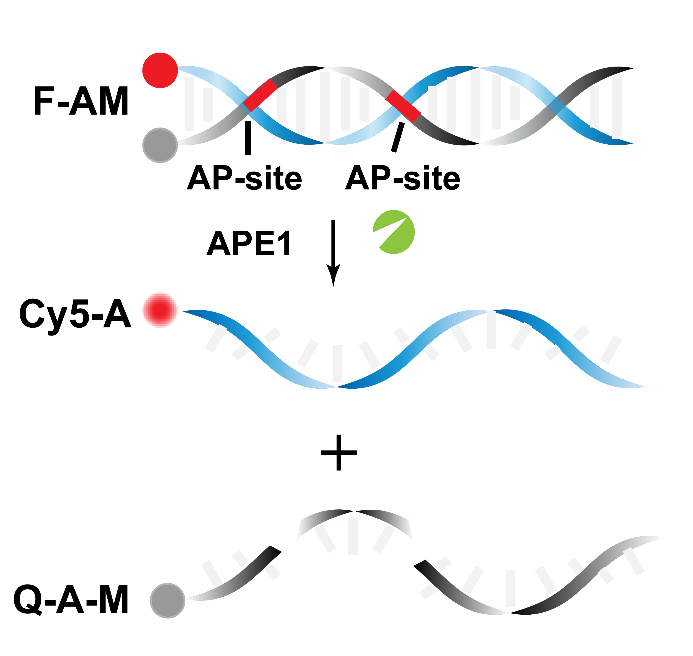


**Figure S1.** The schematic representation of the formation of F-AM via the hybridization of Cy5-A with Q-A-M.


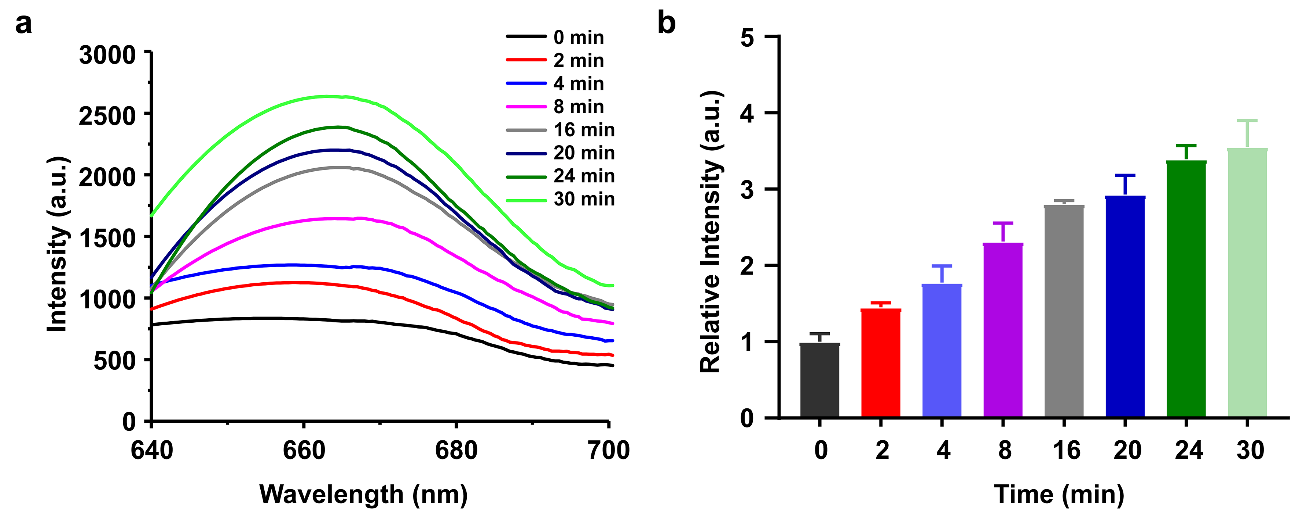


**Figure S2.** Fluorescence spectra (a) and the fluorescence quantification (b) of the F-AM probe (100nm) with APE1 (1 U) for increased times. Data are presented as mean ± SD (n = 3).


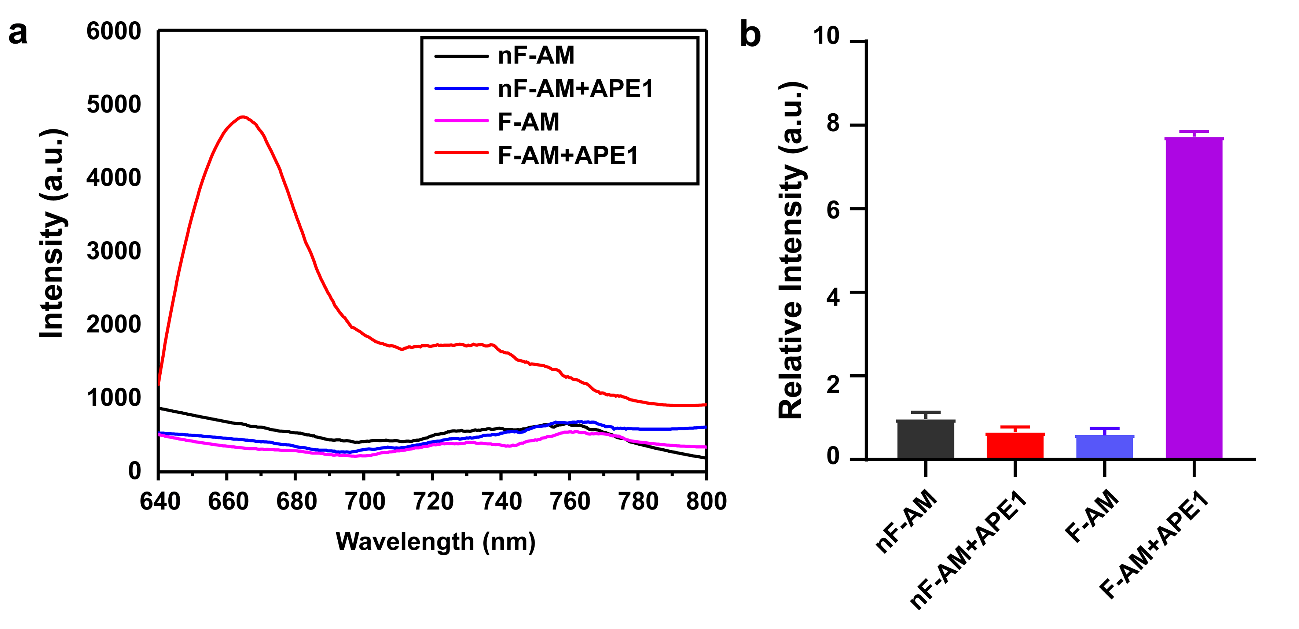


**Figure S3.** Fluorescence spectra (a) and the fluorescence intensity (b) of F-AM (100 nM) and nF-AM with and without APE1. Data are presented as mean ± SD (n = 3).


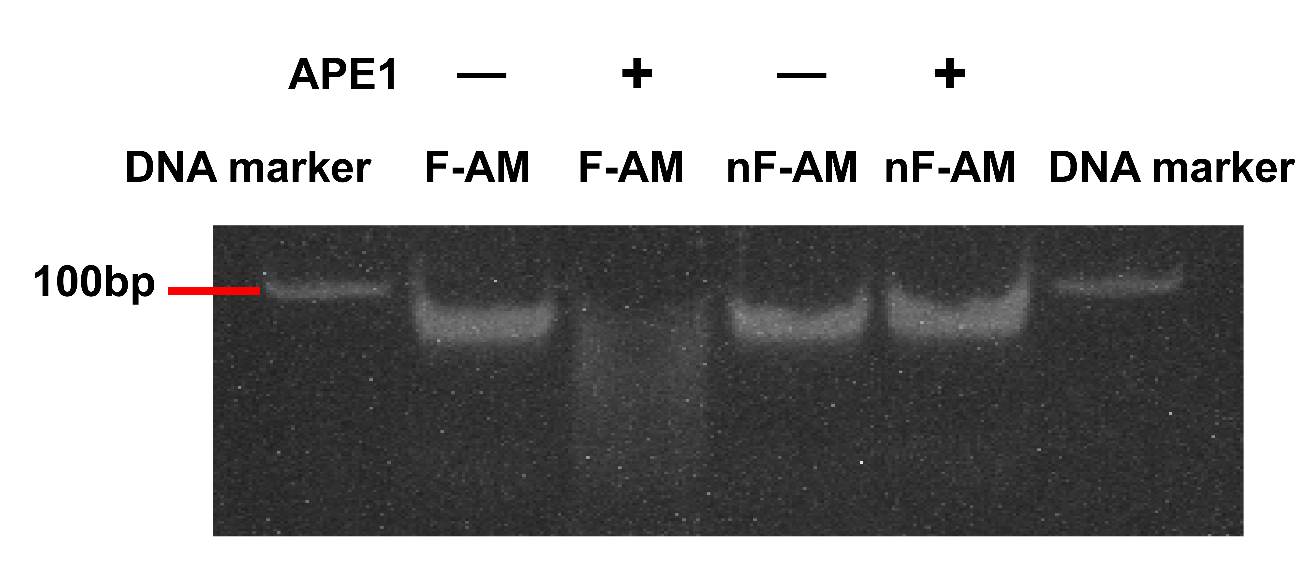


**Figure S4.** PAGE analysis of oligonucleotide damage when F-AM or nF-AM mixed with or without APE1.


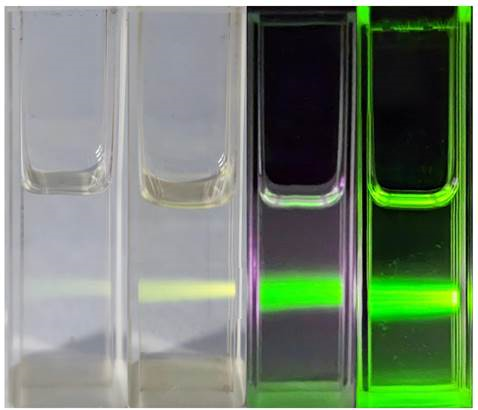


**Figure S5.** The photographs of the solution of NaYF_4_:Yb,Er UCNPs and NaYF_4_:Yb,Er@NaYF_4_ UCNPs in daytime and dark conditions under 980 nm laser illumination (from left to right).


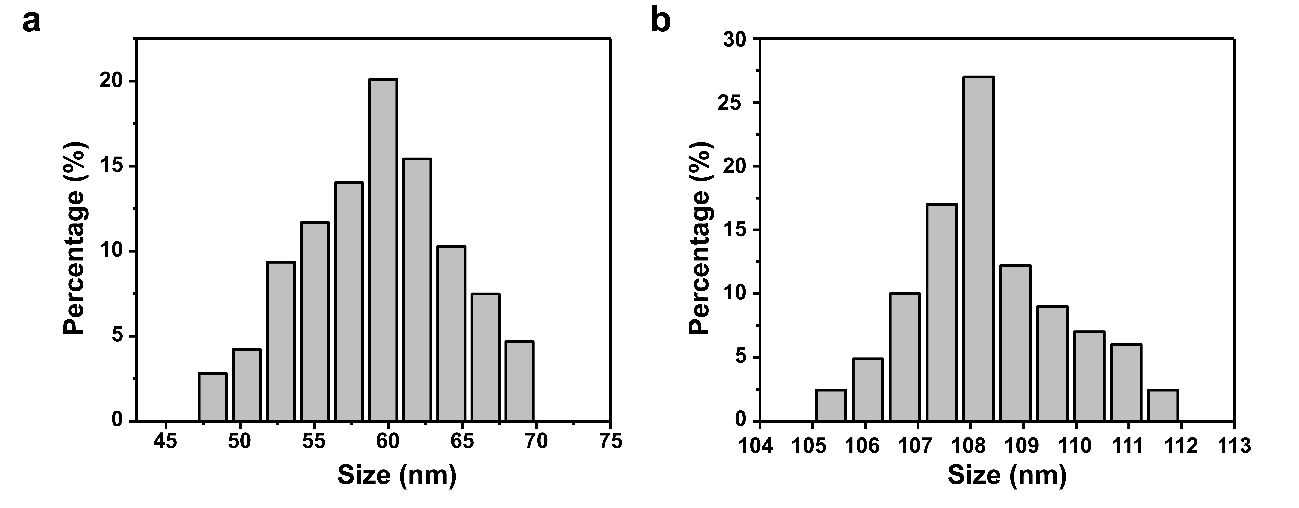


**Figure S6.** The corresponding size distribution of (a) core-shell structured UCNPs and (b) URMT.


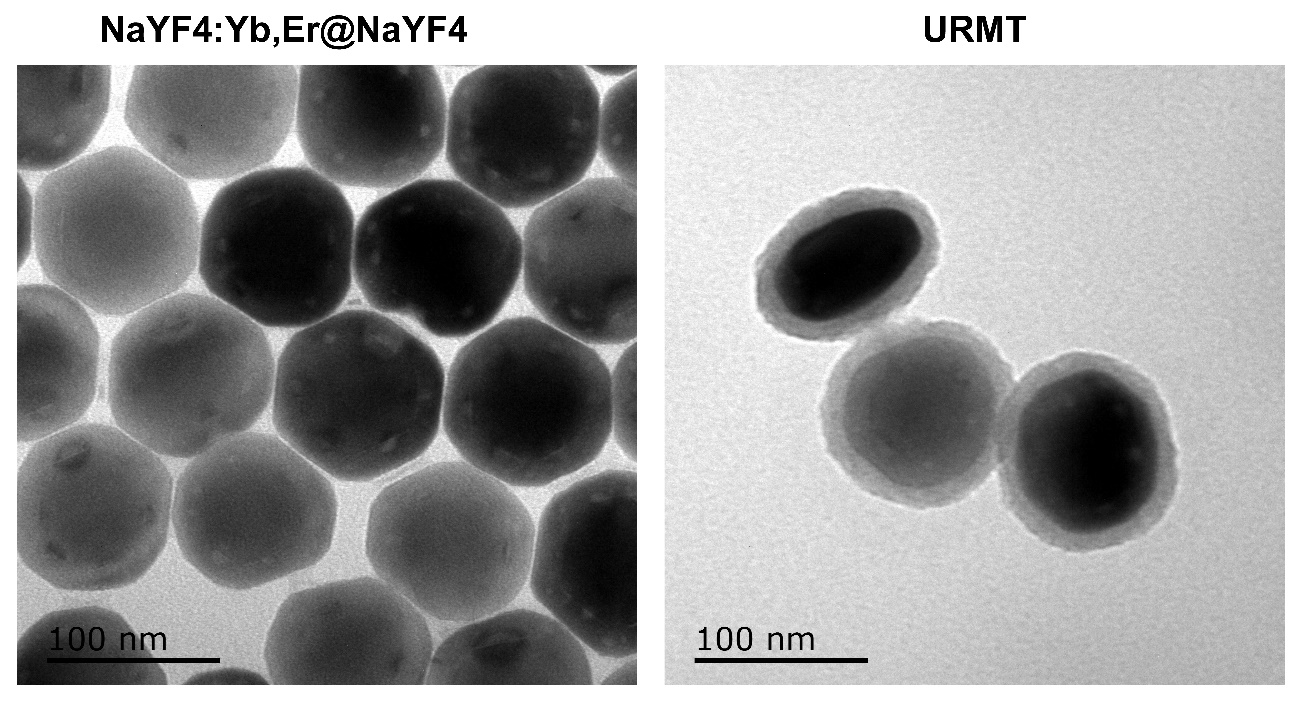


**Figure S7.** TEM images of core-shell structured NaGdF_4_:Yb, Er@NaYF_4_ UCNPs (left) and URMT (right).


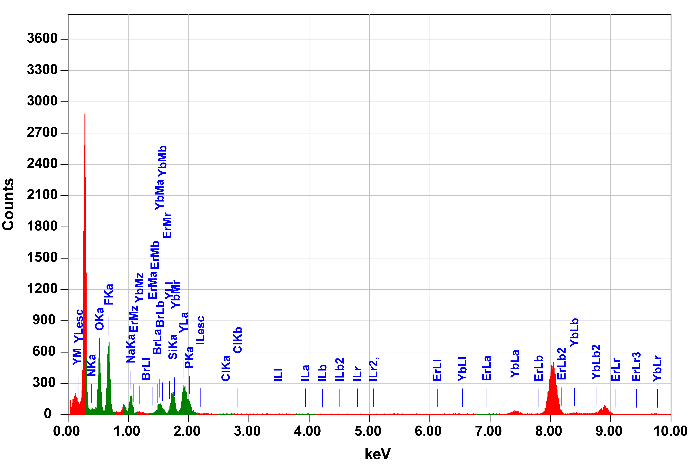


**Figure S8.** EDS mapping of URMT.


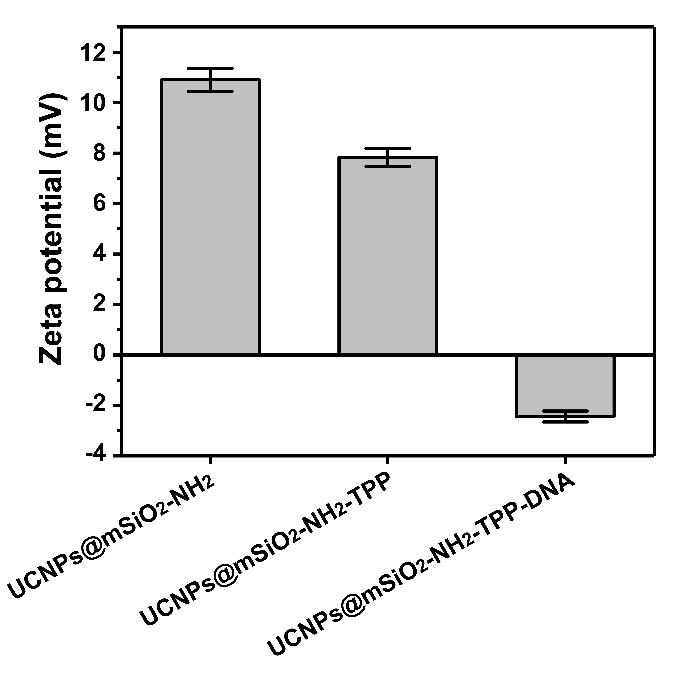


**Figure S9.** Changes in zeta potentials during the synthesis of UCNPs@mSiO_2_-NH_2_, UCNPs@mSiO_2_-TPP, UCNPs@mSiO_2_-TPP-DNA. Data are presented as mean ± SD (n = 3).


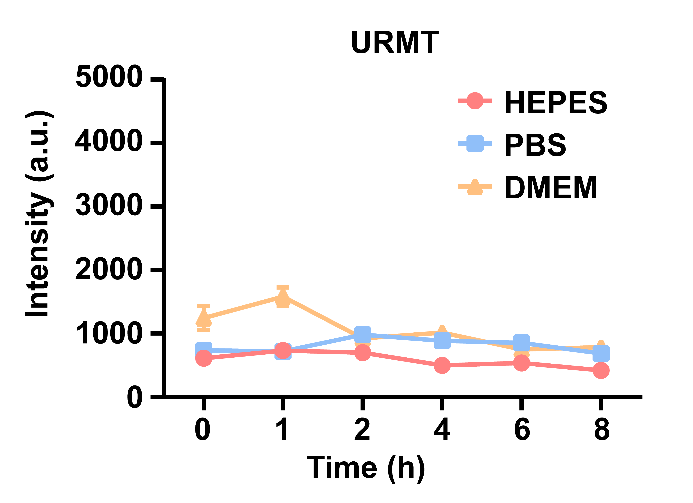


**Figure S10.** Fluorescence intensity of URMT nanoparticle supernatant in HEPES, PBS and DMEM for different time points. Data are presented as mean ± SD (n = 3)


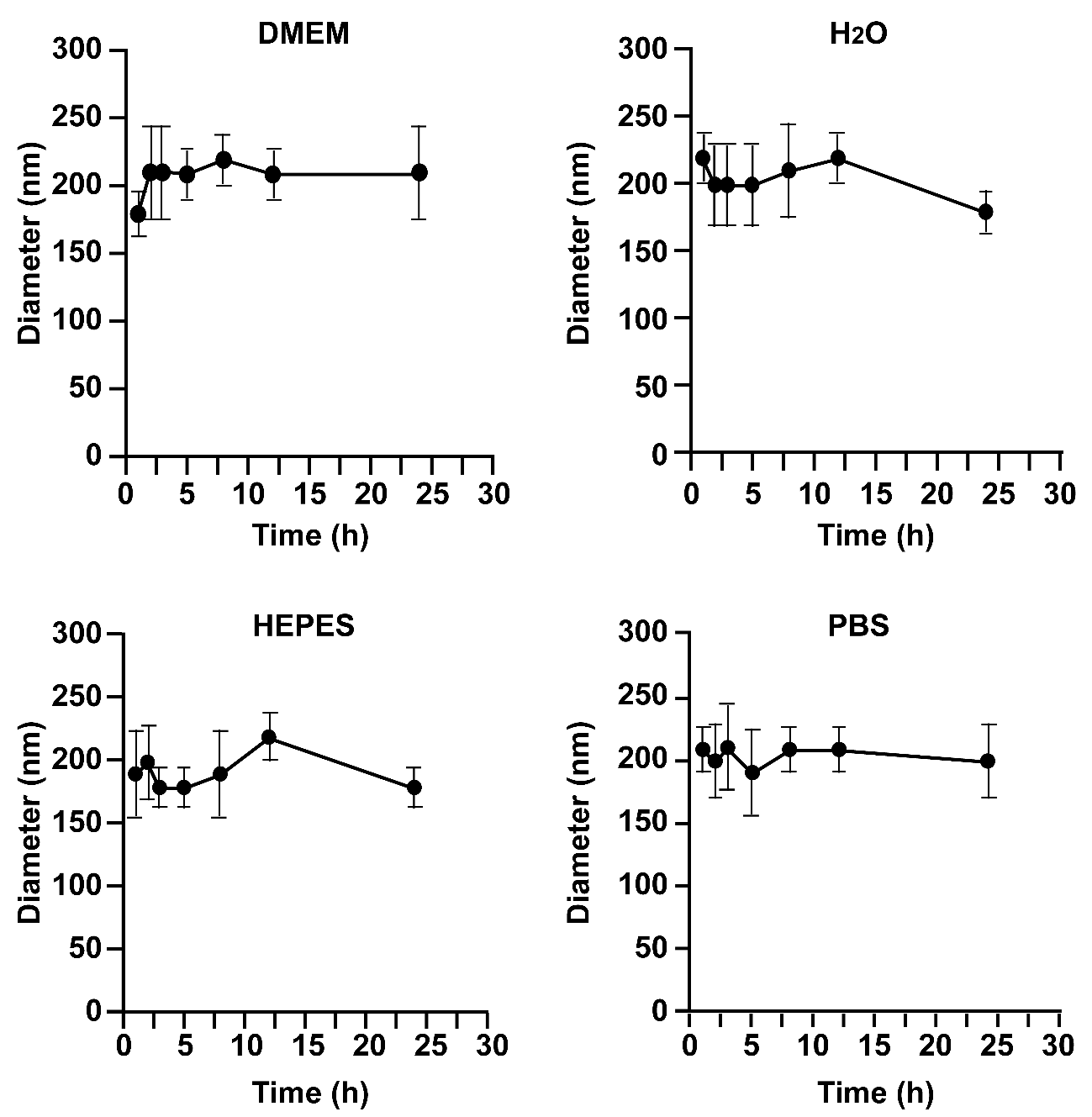


**Figure S11.** Stability of URMT nanoparticle in HEPES, PBS, H_2_O and DMEM at different time points. Data are presented as mean ± SD (n = 3)


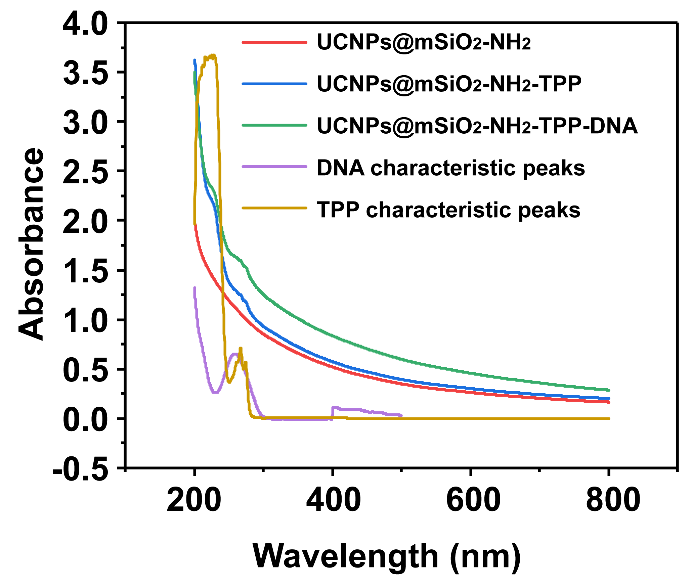


**Figure S12.** UV/Vis absorption spectra of UCNPs@mSiO_2_-NH_2_, UCNPs@mSiO_2_-NH_2_-TPP, and UCNPs@mSiO_2_-NH_2_-DNA-TPP, and characteristic peaks of DNA and TPP.


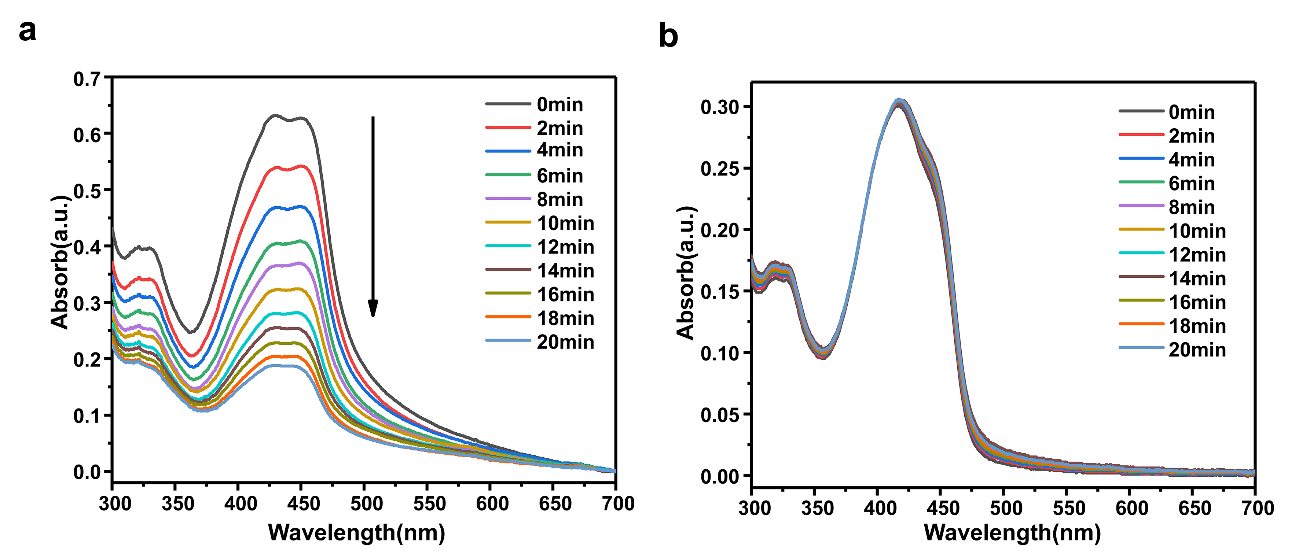


**Figure S13.** ^1^O_2_ generation induced by URMT with (a) and without (b) 980 nm light irradiation determined by time-course of absorption decrease of DPBF probe.


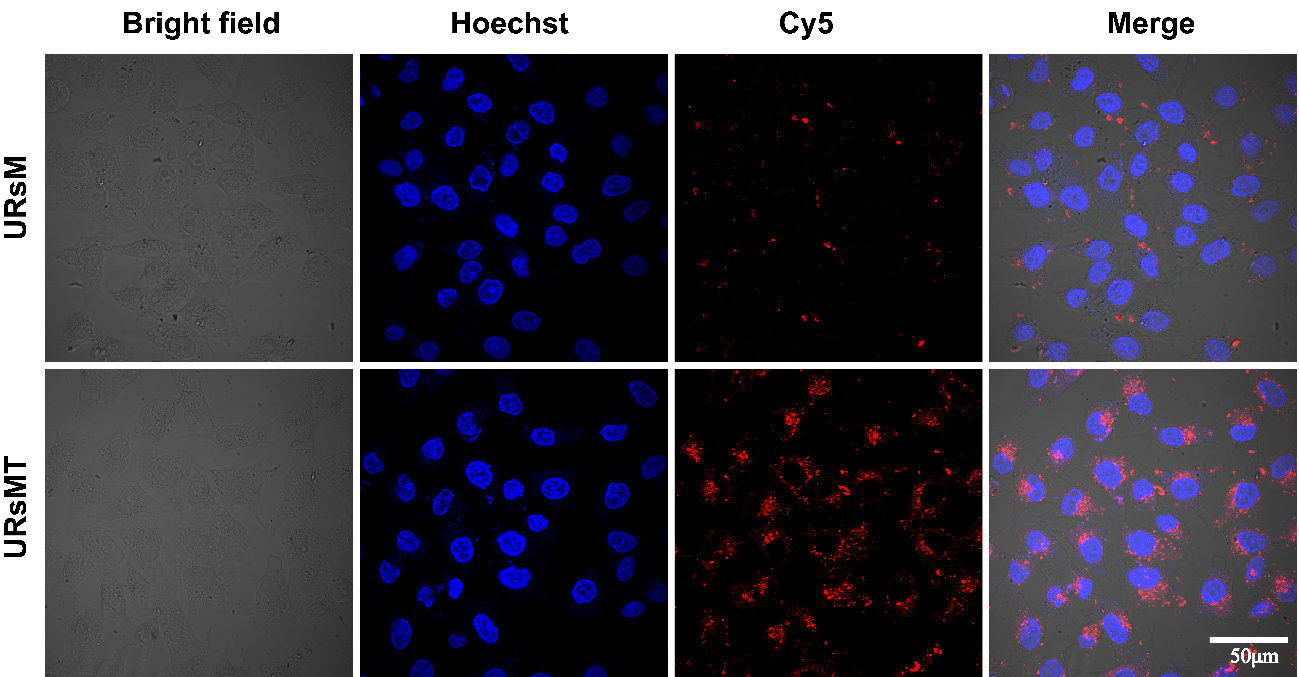


**Figure S14.** CLSM images of MCF-7 cells treated with URM and URMT (miR21 was labeled with Cy5) and and Hoechst 33342 (blue). Scale bar, 50 μm.


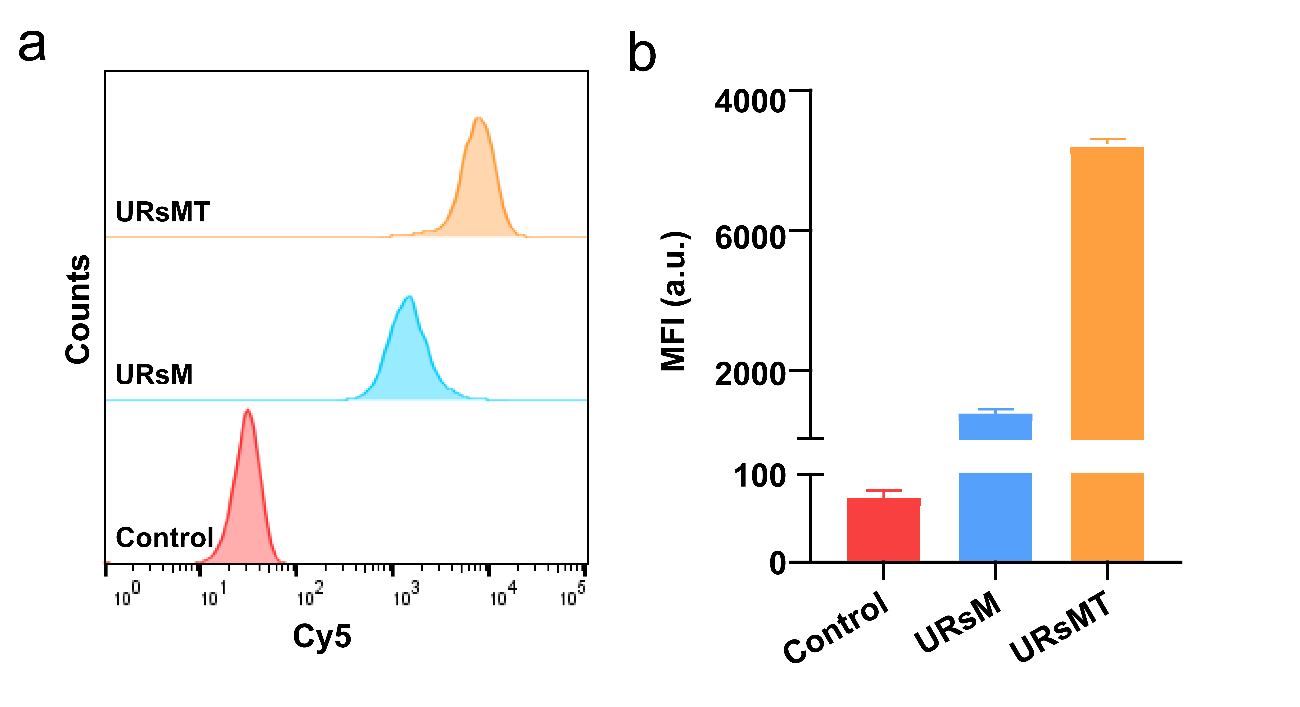


**Figure S15.** (a) Flow cytometry showing the fluorescence of MCF-7 cells with URsM and URsMT (where sM represents the single stranded anti-miR21 labeled with Cy5). The quantification of the flow cytometric data in (b). Data are presented as mean ± SD (n = 3).


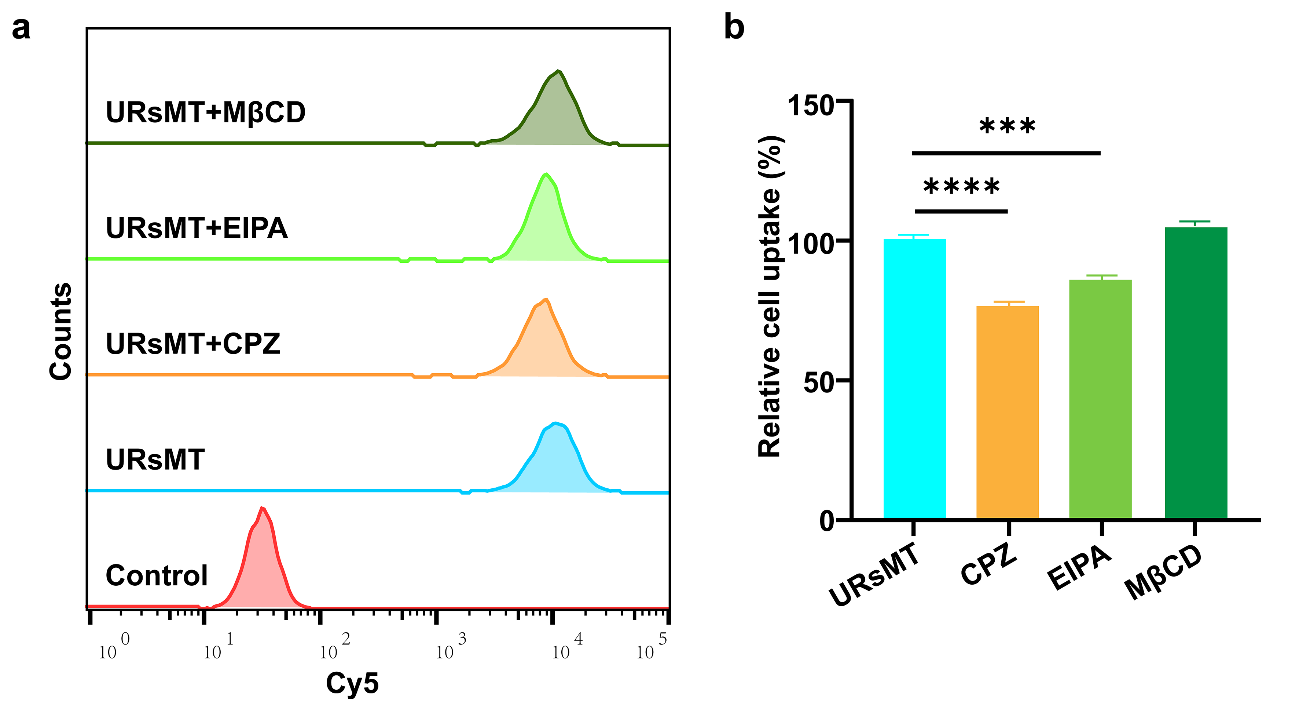


**Figure S16.** (a) Flow cytometry analysis of cellular uptake efficiency of URsMT nanoparticles pre-treated with various endocytosis inhibitors. (b) The quantification of the flow cytometric data. Chlorpromazine:CPZ; 5-(N-ethyl-N-isopropyl: EIPA; Methyl-β-cyclodextrin: MβCD. ****P* < 0.001, *****P* < 0.0001. Data are presented as mean ± SD (n = 3).


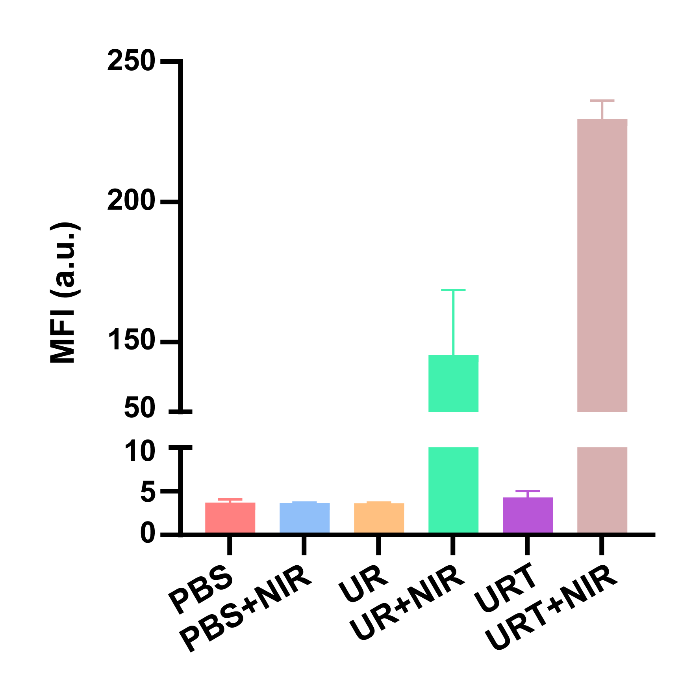


**Figure S17.** The quantities analysis of ROS production in MCF-7 cells by CLSM. Data are presented as mean ± SD (n = 3).


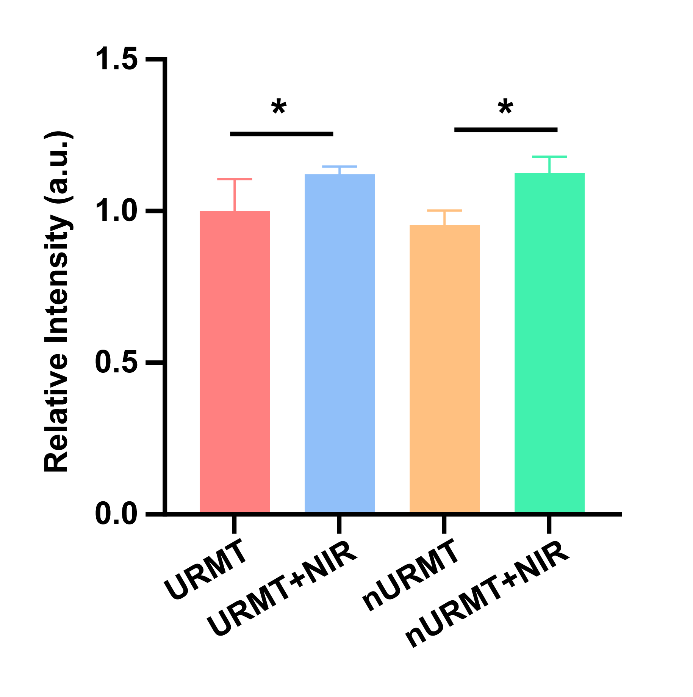


**Figure S18.** The quantities analysis of APE1 immunofluorescence intensity on the CLSM images. **P* < 0.05. Data are presented as mean ± SD (n = 3).


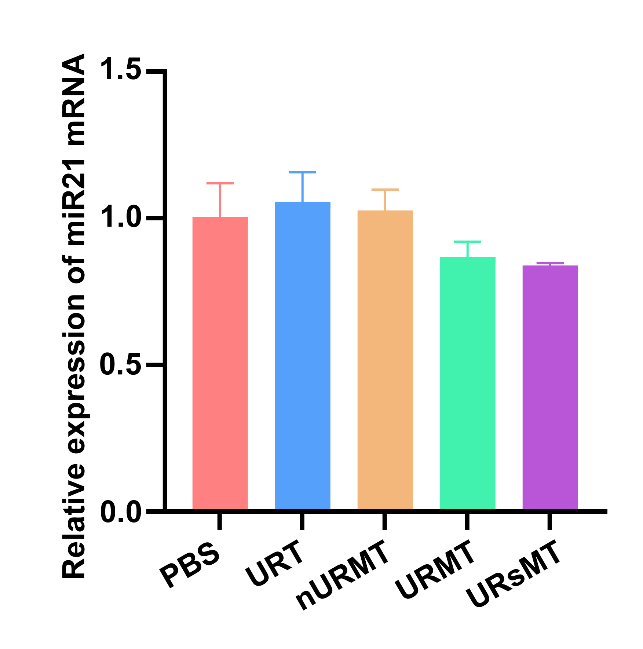


**Figure S19.** qRT-PCR analysis of the mRNA expression of miR21 in MCF-7 cells without NIR irradiation upon different treatments. Data are presented as mean ± SD (n = 3).


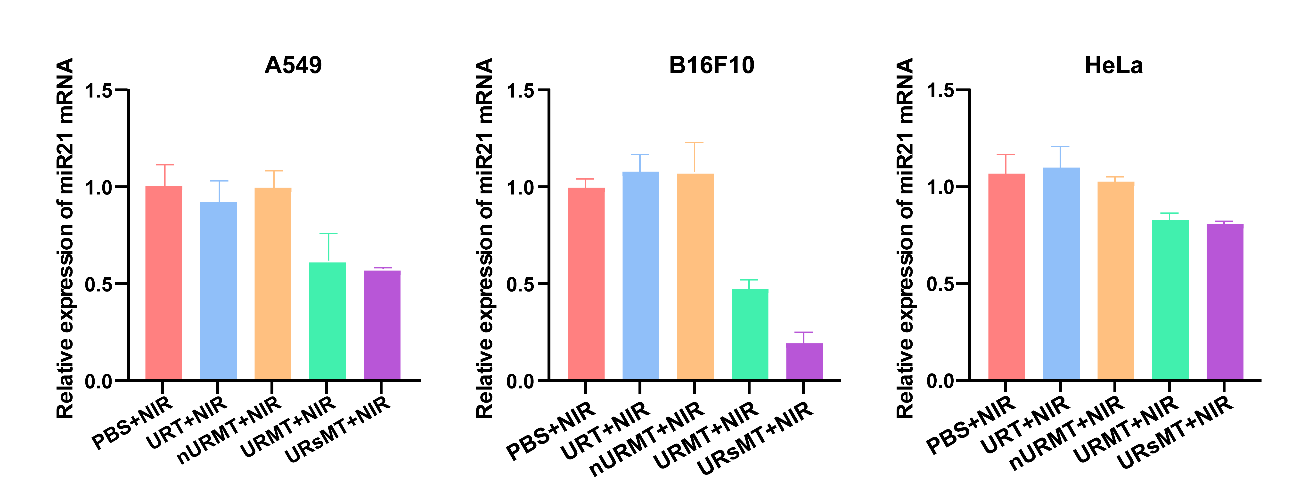


**Figure S20.** qRT-PCR analysis of the mRNA expression of miR21 in A549, B16F10 and HeLa cells upon different treatments. Data are presented as mean ± SD (n = 3).


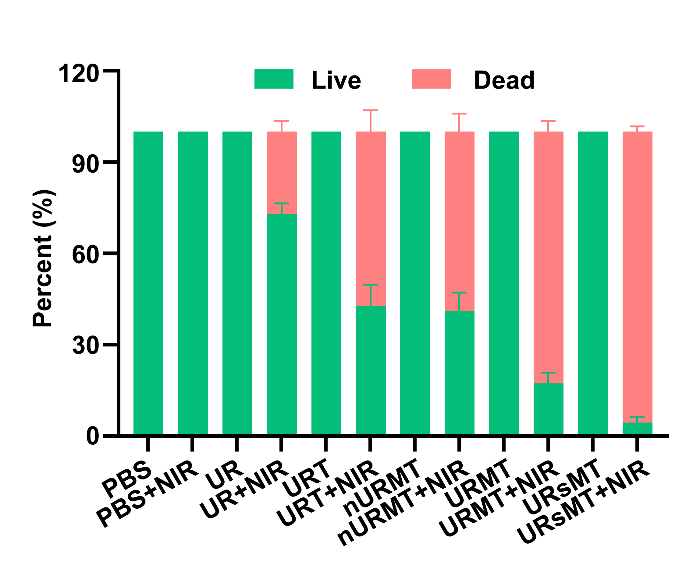


**Figure S21.** The quantities analysis of live/dead double staining by CLSM. Data are presented as mean ± SD (n = 3).


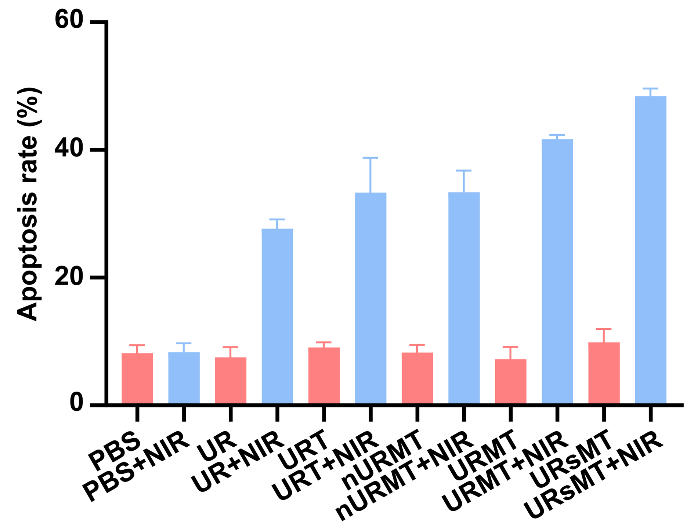


**Figure S22.** Cell apoptosis analyzed by flow cytometry with different treatments. Data are presented as mean ± SD (n = 3).


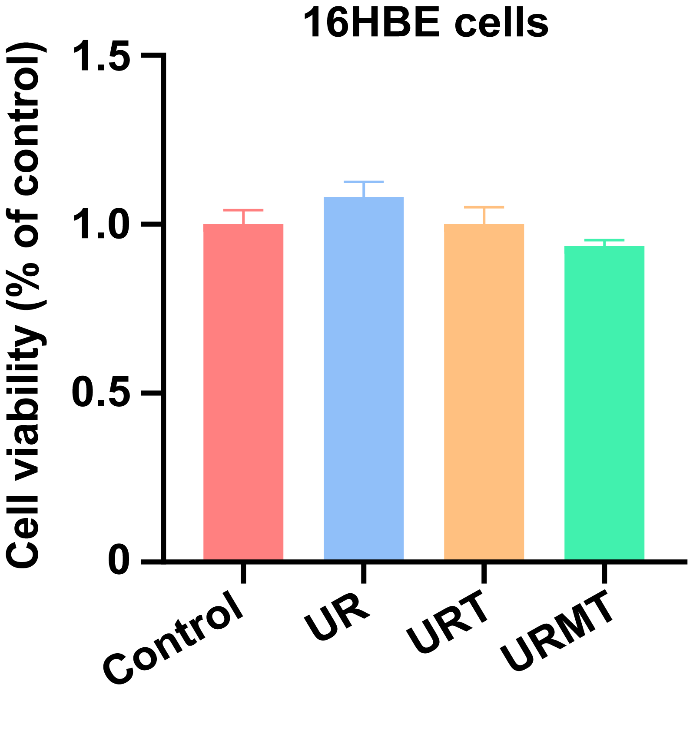


**Figure S23.** Cell viability of 16HBE cells incubated with different treatments in the dark. Data are presented as mean ± SD (n = 3).


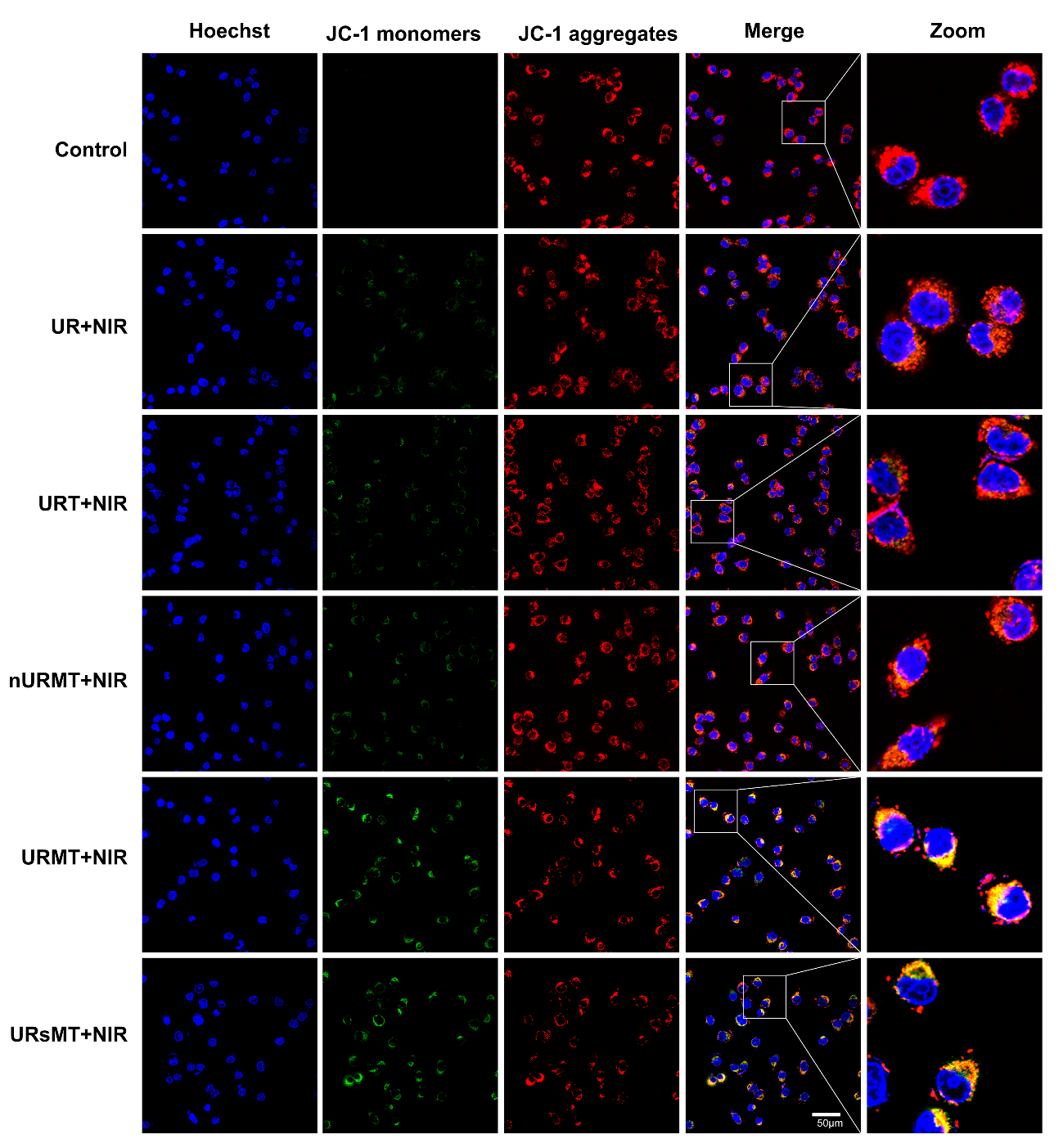


**Figure S24.** Mitochondrial membrane potential of MCF-7 cells treated with UR, URT, nURMT, URMT and URsMT with laser irradiation. The scale bars in the last images apply to the others in the same panels.


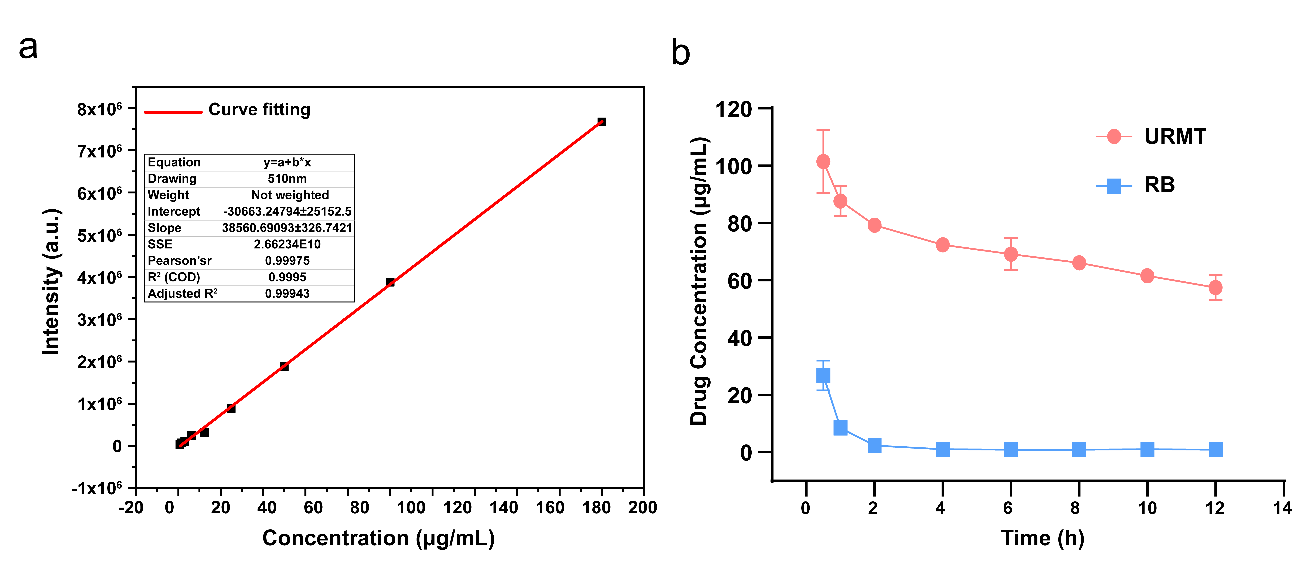


**Figure S25.** *In vivo* pharmacokinetics of URMT nanoparticles. (a): The standard·curve of RB. (b) The plasma drug concentrations of free drug in BALB/c mice after injection of RB (1.6 mg/kg) or URMT nanoparticles (containing RB 1.6 mg/kg) at different time points measured by fluorescence spectroscopy and HPLC. Data are presented as mean ± SD (n = 3).


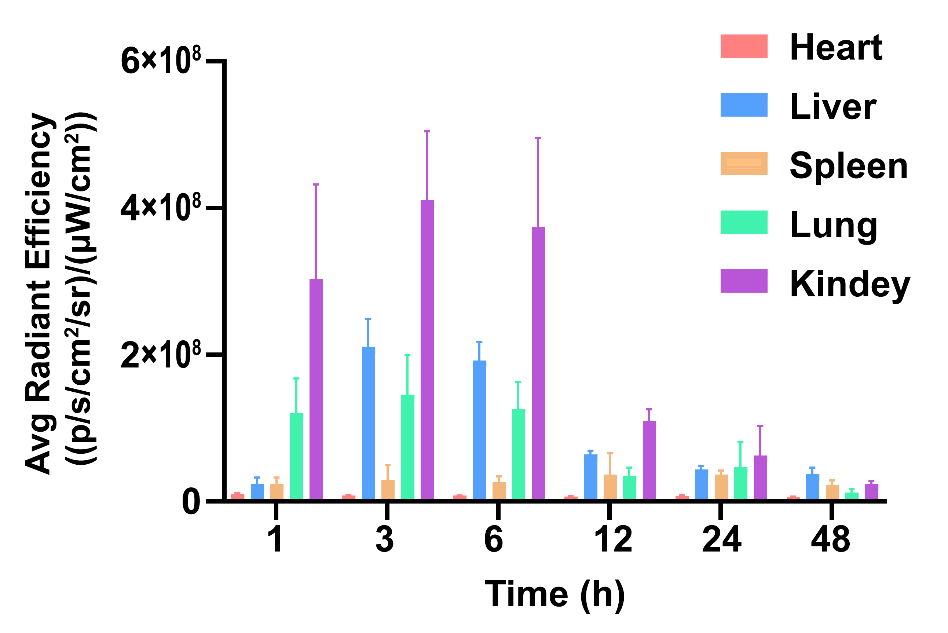


**Figure S26.** The quantitative analysis of anti-miR21 fluorescence intensity in different organs. Data are presented as mean ± SD (n = 3).


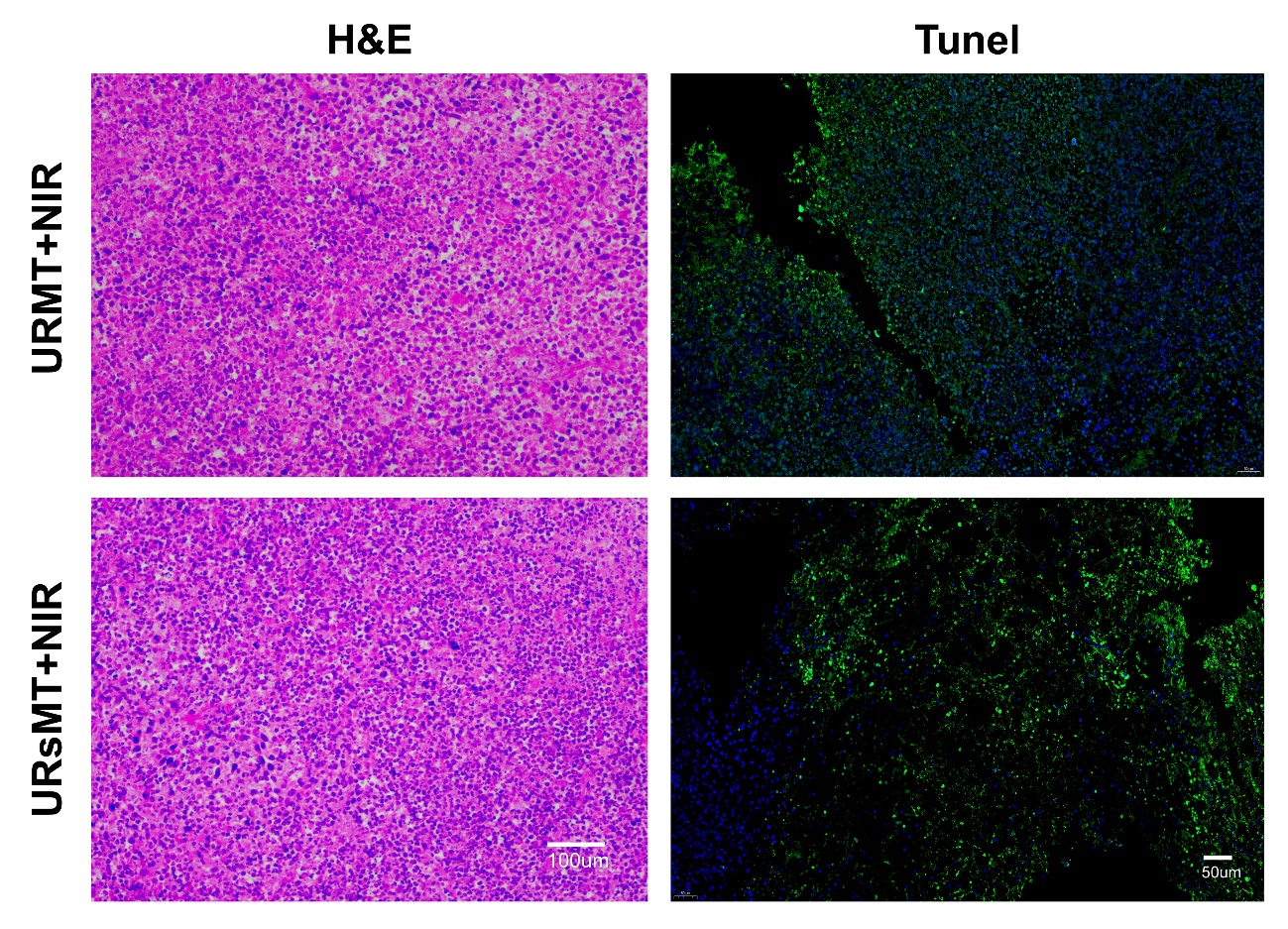


**Figure S27.** H&E (left) and TUNEL (right) staining of the tumor sections with URMT +NIR and UrsMT + NIR.


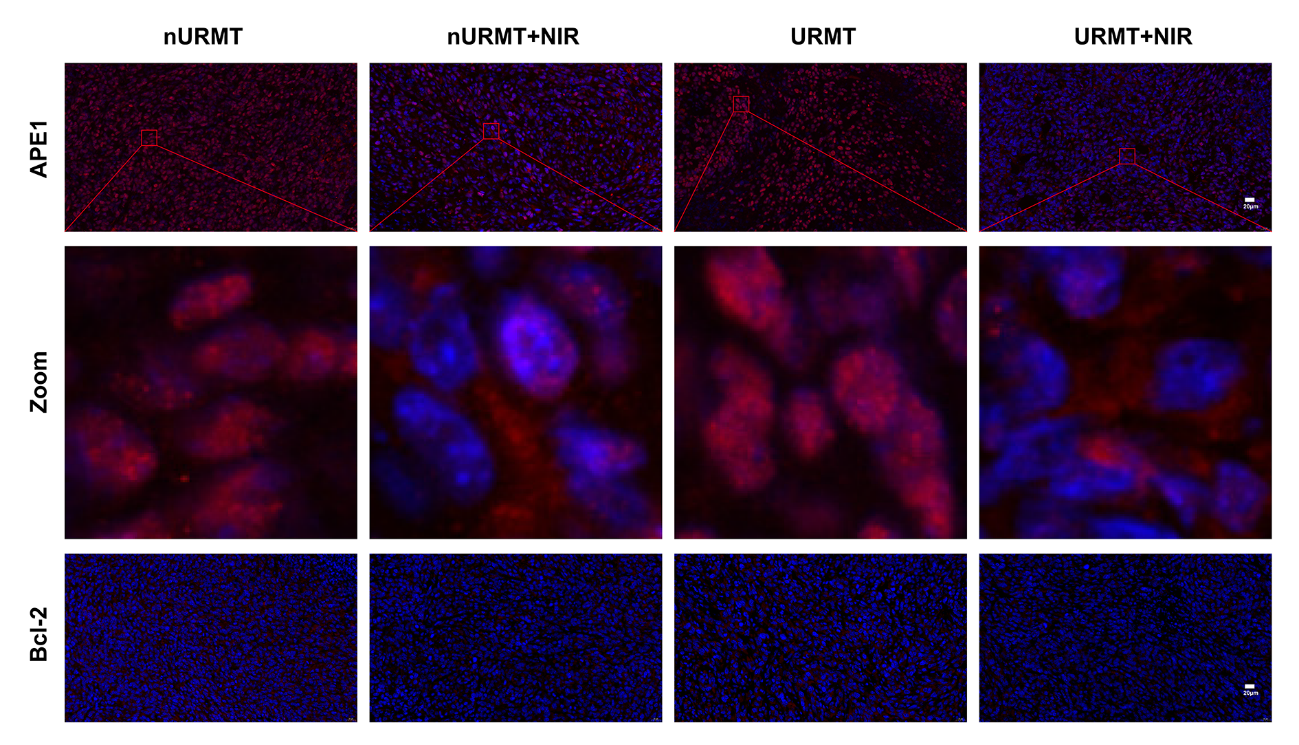


**Figure S28.** Representative immunofluorescence images of APE1 and Bcl-2 staining sections of tumor tissues from different treatment groups. Scale bar: 20 μm.


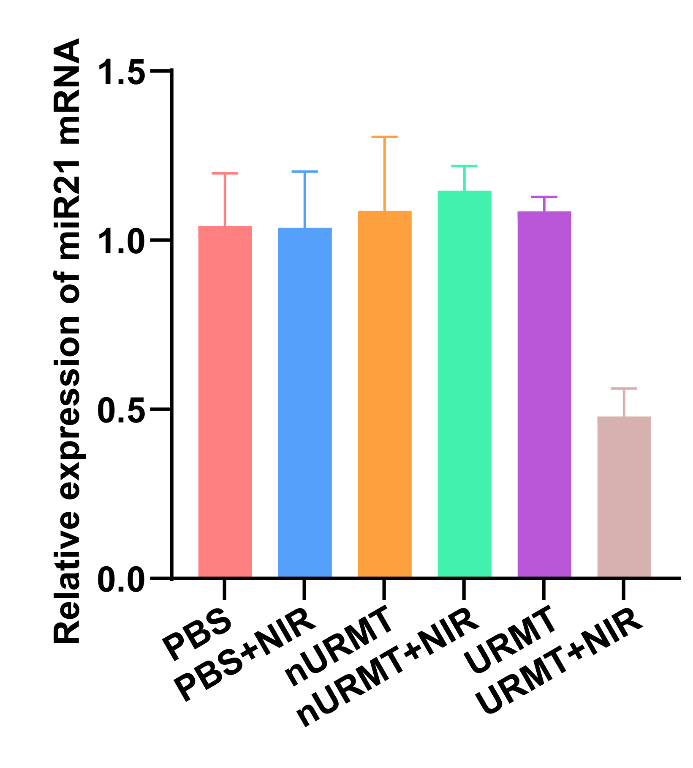


**Figure S29.** qRT-PCR analysis of the mRNA expression of miR21 in tumor upon different treatments. Data are presented as mean ± SD (n = 3).


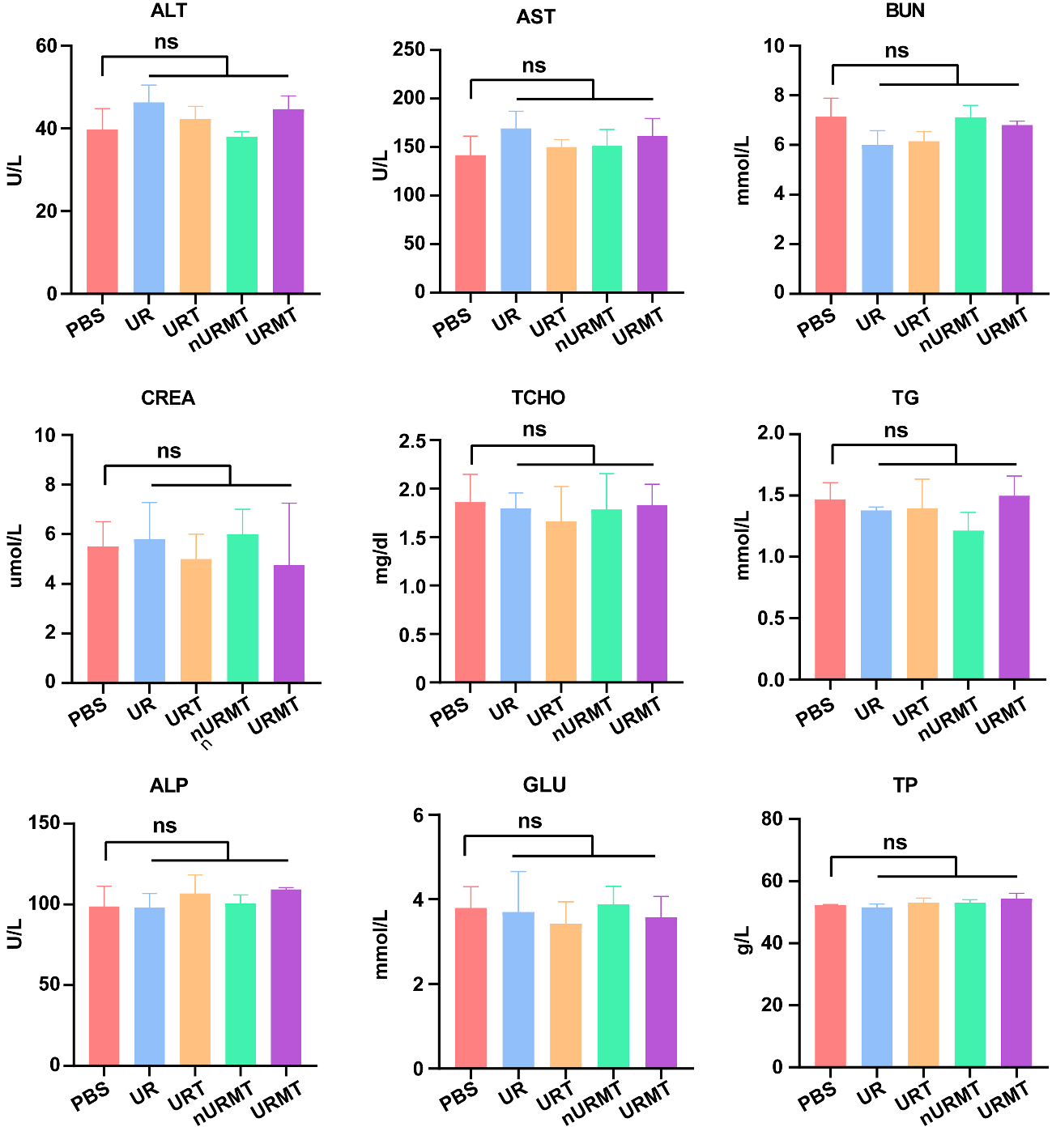


**Figure S30.** Blood biochemical assay of ALT, AST, BUN, CREA, TCHO, TG, ALP, GLU and TP from the mice after different treatments, respectively. Data are presented as mean ± SD (n = 4). ns = no significance.


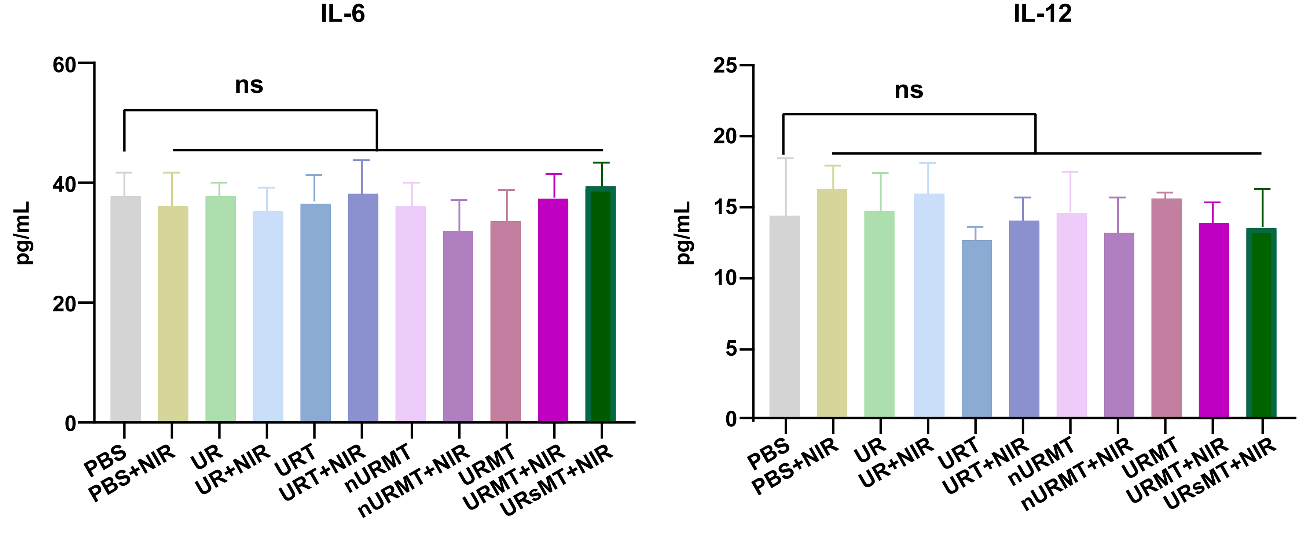


**Figure S31.** The inflammatory cytokines expression of IL-6 and IL-12 after different treatments. Data are presented as mean ± SD (n = 4). ns = no significance.


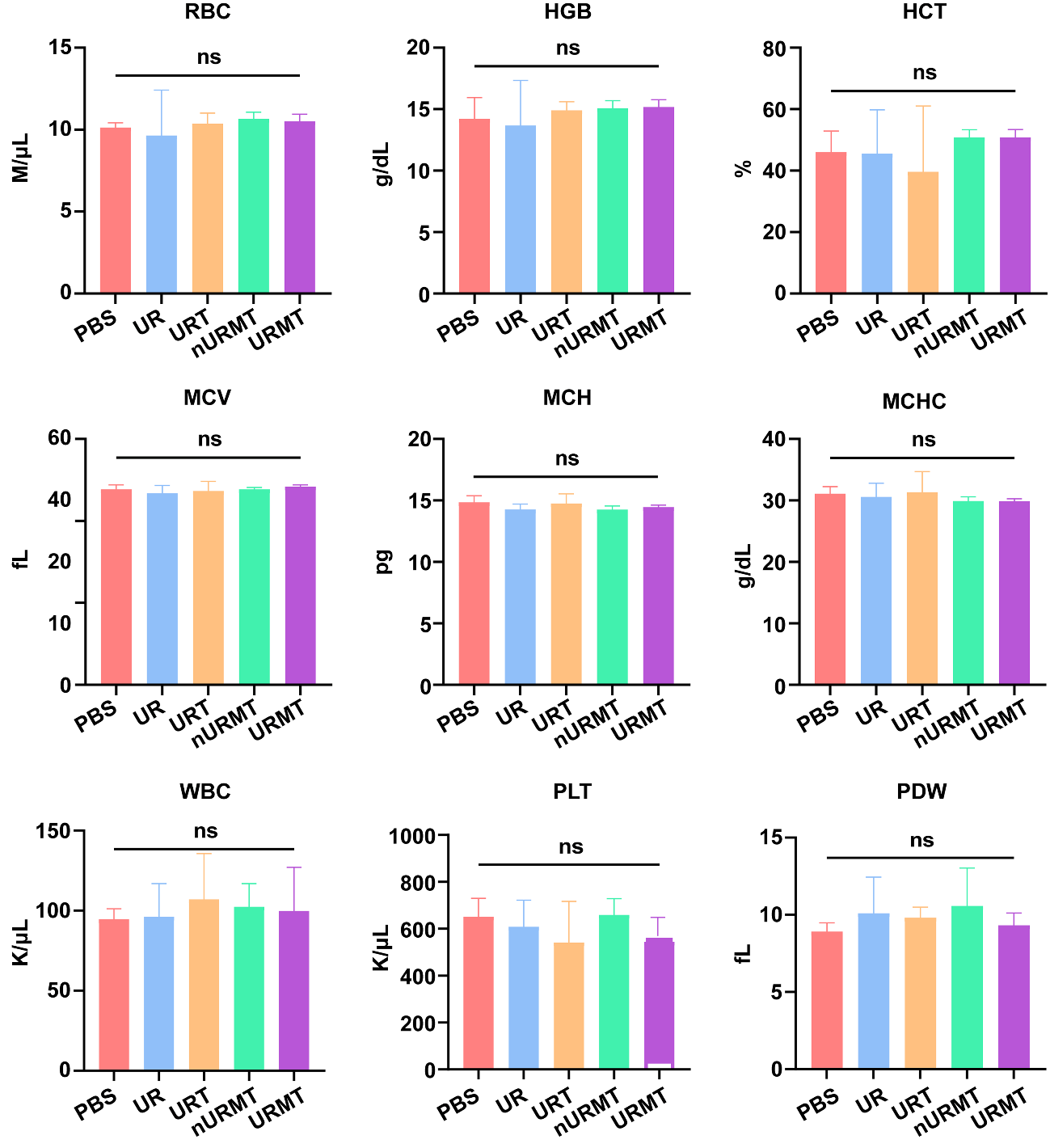


**Figure S32.** Blood routine assessment (red blood cell count: RBC, hemoglobin: HGB, hematocrit: HCT, mean corpuscular volume: MCV, mean corpuscular hemoglobin: MCH, mean corpuscular hemoglobin concentration: MCHC, white blood cell count: WBC, platelet: PLT, platelet distribution width: PDW) to estimate the effects of different groups to blood cells. Data are presented as mean ± SD (n = 4). ns = no significance.


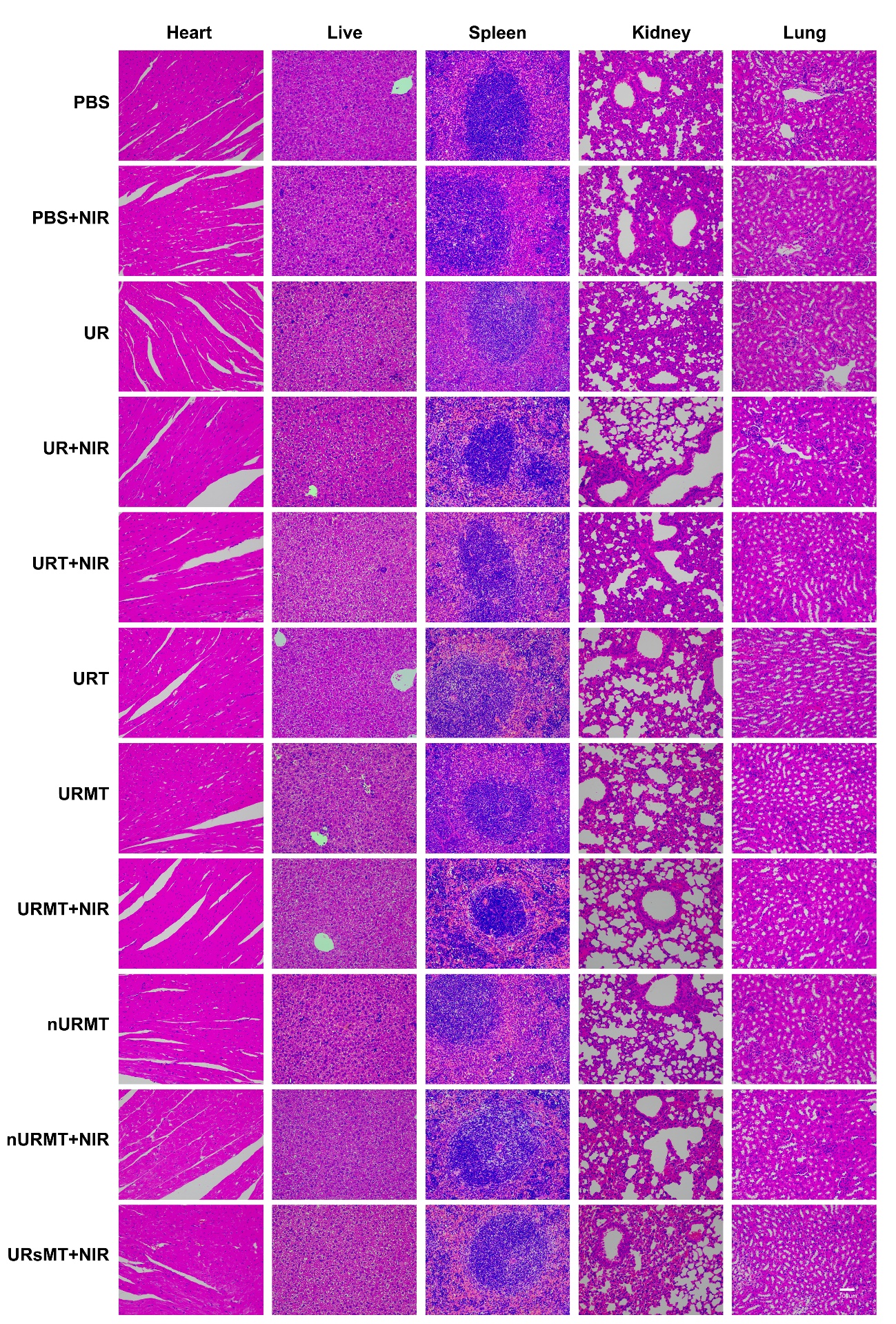


**Figure S33.** Representative H&E-stained tissue sections from different treatments mice. Scale bar, 100 µm.


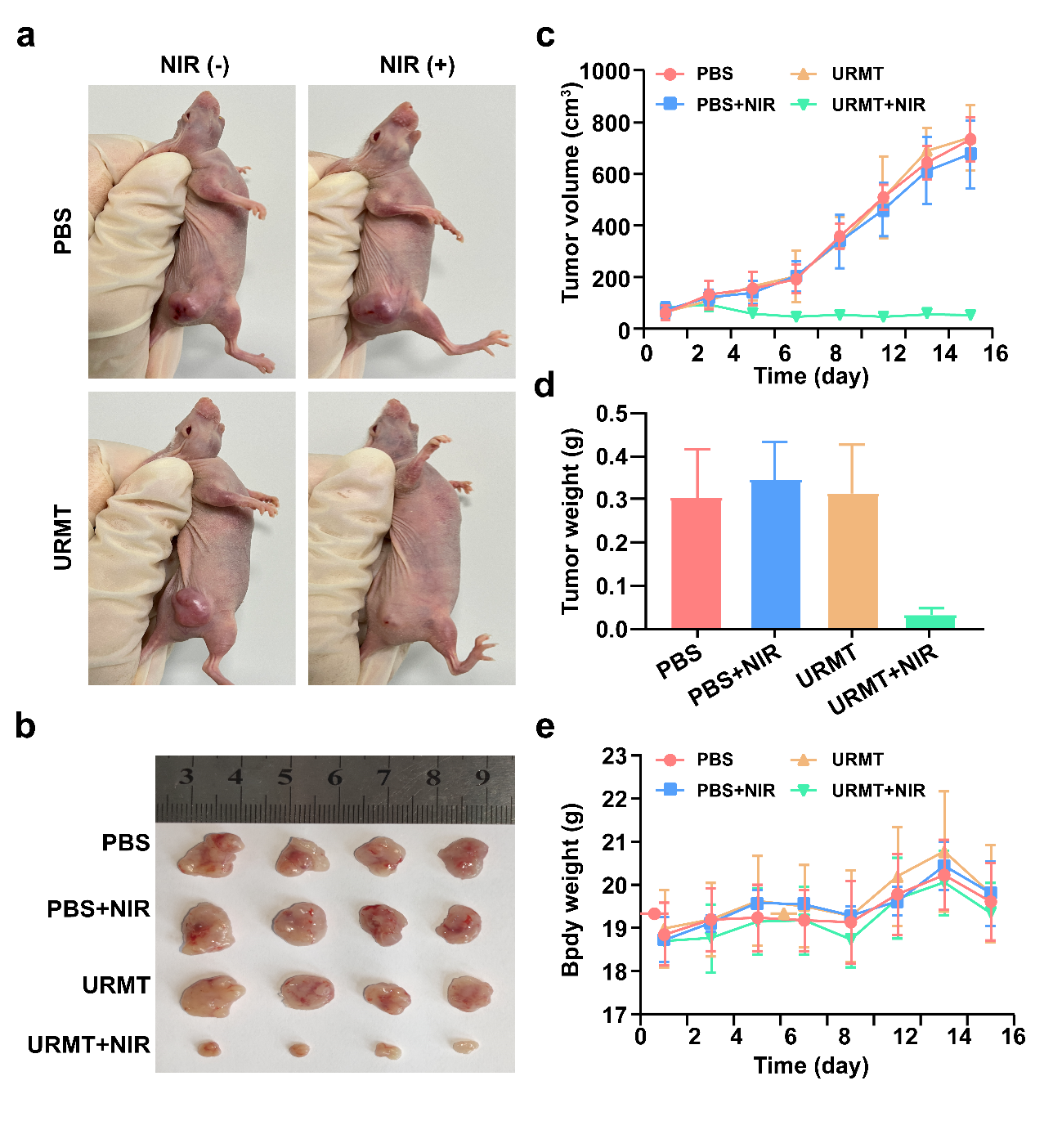


**Figure S34.** URMT induces an enhanced antitumor effect in MCF-7 tumor bearing nude mice. (a) Body photographs. (b) Tumor photographs. (c) Tumor growth curves and (d) tumor weight in MCF-7 tumor bearing mice after different treatments. (e) Body weight of MCF-7 tumor-bearing mice in the different treatments. Data are presented as mean ± SD (n = 4).

**Table S1**. The quantitative analysis of each element in URMT

| **Element** | **Na** | **Y** | **F** | **Yb** | **Er** | **Si** | **O** | **N** | **P** | **Cl** | **Br** | **I** | **Total** |
| --- | --- | --- | --- | --- | --- | --- | --- | --- | --- | --- | --- | --- | --- |
| **Mass%** | 4.55 | 40.47 | 19.92 | 8.9 | ND | 4.52 | 19.06 | 1 | ND | ND | 1.59 | ND | 100 |
| **Atom%** | 6.19 | 14.24 | 32.8 | 1.61 | ND | 5.03 | 37.27 | 2.23 | ND | ND | 0.62 | ND | 100 |

**Table S2**. The sequence of DNA used in this study

| Name | Sequence (5' - 3') |
| --- | --- |
| miR-21 | TAGCTTATCAGACTGATGTTGA |
| anti-miR21 | TCAACATCAGTCTGATTAGCTA |
| miR21-AP | TAGCTT/ids p/TCAGACTG/ids p/TGTTGA |
| miR21 RT primer | CTCAACTGGTGTCGTGGAGTCGGCAATTCAGTTGAGTCAACATCA |
| miR21 Forword primer mouse | GGACTAGCTTATCAGACTG |
| miR21 Reverse primer mouse | CATCAGATGCGTTGCGTA |
| miR21 Forword primer human | TCACCTTCACCGTTCCAGTTT |
| miR21 Reverse primer human | TGGTGTCGTGGAGTCG |
| U6 RT primer | GTCGTATCCAGTGCAGGGTCCGAGGTATTCGCACTGGATACGACAAAATA |
| U6 Forword primer mouse | ATTGGAACGATACAGAGAAGAT |
| U6 Reverse primer mouse | GGAACGCTTC ACGAATTT |
| U6 Forword primer human | AGAGAAGATTAGCATGGCCCCTC |
| U6 Reverse primer human | ATCCAGTGCAGGGTCCGAGG |

**Table S3.** Full name-abbreviation contrast table

| **Full name** | **Abbreviation** | **Full name** | **Abbreviation** |
| --- | --- | --- | --- |
| 1- octadecene | ODE | near-infrared | NIR |
| 1,3-diphenylisobenzofuran | DPBF | oleic acid | OA |
| 2,7-dichlorofluorescein-diacetate | DCFH-DA | oleylamine | OM |
| alkaline phosphatase | ALP | oxygen | ^1^O_2_ |
| apurinic/apyrimidinic endonuclease 1 | APE1 | phosphate buffered saline | PBS |
| black hole quencher-3 | BHQ3 | Photodynamic therapy | PDT |
| blood glucose | GLU | photosensitizers | PSs |
| blood urea nitrogen | BUN | platelet | PLT |
| Cell Counting Kit-8 | CCK8 | reactive oxygen species | ROS |
| confocal laser scanning microscopy | CLSM | Real-time fluorescence quantitative polymerase chain reaction | qRT-PCR |
| creatinine | CREA | red blood cell count | RBC |
| cyanine5 | Cy5 | Rose Bengal | RB |
| dulbecco's modified eagle medium | DMEM | scanning electron microscope | SEM |
| energy dispersive spectrometer | EDS | TdT-mediated dUTP Nick-End Labeling | TUNEL |
| fetal bovine serum | FBS | total cholesterol | TCHO |
| Förster resonance energy transfer | FRET | total protein | TP |
| Fourier transform infrared | FTIR | Transmission electron microscopy | TEM |
| glutamic oxalacetic transaminase | AST | triglyceride | TG |
| glutamic-pyruvic transaminase | ALT | triphenylphosphine | TPP |
| hematocrit | HCT | UCNP@mSiO_2_-RB-TPP-DNA (double strands hybridizing an antisense oligonucleotide (anti-miR21) with a complementary DNA (miR21) containing basic AP sites) | URMT |
| hematoxylin and eosin | H&E | UCNP@mSiO_2_-RB-TPP-DNA (double strands hybridizing an antisense oligonucleotide (anti-miR21) with a complementary DNA (miR21) without basic AP site) | nURMT |
| hemoglobin | HGB | UCNPs@mSiO_2_-RB | UR |
| High Performance Liquid Chromatography | HPLC | UCNPs@mSiO_2_-RB-anti-miR21(loaded with a single strand of anti-miR21) | URsM |
| *in vivo* imaging system | IVIS | UCNPs@mSiO_2_-RB-TPP | URT |
| mean corpuscular hemoglobin | MCH | UCNPs@mSiO_2_-RB-TPP-anti-miR21(loaded with a single strand of anti-miR21) | URsMT |
| mean corpuscular hemoglobin concentration | MCHC | ultraviolet | UV |
| mean corpuscular volume | MCV | upconversion nanoparticles | UCNPs |
| messenger RNA | mRNA | upconversion nanoparticles | UCNPs |
| MicroRNAs | miRNAs | visible | Vis |
| mitochondria membrane potential | MMP | white blood cell count | WBC |
| mitochondria membrane potential | MMP | X-ray diffraction | XRD |
